# Gold Nanostrip Array‐Mediated Wireless Electrical Stimulation for Accelerating Functional Neuronal Differentiation

**DOI:** 10.1002/advs.202202376

**Published:** 2022-05-26

**Authors:** Hongru Yang, Yue Su, Zhaoyang Sun, Baojin Ma, Feng Liu, Ying Kong, Chunhui Sun, Boyan Li, Yuanhua Sang, Shuhua Wang, Gang Li, Jichuan Qiu, Chao Liu, Zhaoxin Geng, Hong Liu

**Affiliations:** ^1^ State Key Laboratory of Crystal Materials Shandong University Jinan Shandong 250100 P. R. China; ^2^ School of Information Engineering Minzu University of China Beijing 100081 P. R. China; ^3^ Department of Oral and Maxillofacial Surgery Qilu Hospital of Shandong University Jinan Shandong 250012 P. R. China; ^4^ Institute for Advanced Interdisciplinary Research University of Jinan Jinan Shandong 250022 P. R. China; ^5^ State Key Laboratory of Integrated Optoelectronics Institute of Semiconductors Chinese Academy of Sciences Beijing 100083 P. R. China; ^6^ Department of Neurosurgery Qilu Hospital Cheeloo College of Medicine and Institute of Brain and Brain‐Inspired Science Shandong University Jinan Shandong 250012 P. R. China

**Keywords:** electromagnetic induction, mature functional neuron, neuronal differentiation, NSC‐based therapy, wireless electrical stimulation

## Abstract

Neural stem cell (NSC)‐based therapy holds great promise for the treatment of neurodegenerative diseases. Presently, however, it is hindered by poor functional neuronal differentiation. Electrical stimulation is considered one of the most effective ways to promote neuronal differentiation of NSCs. In addition to surgically implanted electrodes, traditional electrical stimulation includes wires connected to the external power supply, and an additional surgery is required to remove the electrodes or wires following stimulation, which may cause secondary injuries and infections. Herein, a novel method is reported for generation of wireless electrical signals on an Au nanostrip array by leveraging the effect of electromagnetic induction under a rotating magnetic field. The intensity of the generated electrical signals depends on the rotation speed and magnetic field strength. The Au nanostrip array‐mediated electric stimulation promotes NSC differentiation into mature neurons within 5 days, at the mRNA, protein, and function levels. The rate of differentiation is faster by at least 5 days than that in cells without treatment. The Au nanostrip array‐based wireless device also accelerates neuronal differentiation of NSCs in vivo. The novel method to accelerate the neuronal differentiation of NSCs has the advantages of wireless, timely, localized and precise controllability, and noninvasive power supplementation.

## Graphical abstract

This work describes the acceleration of neural stem cell (NSC) differentiation into mature, functional neurons within 5 days using electrical stimulation generated on an Au nanostrip array under a rotating magnetic field through the electromagnetic induction effect, thereby providing a novel method to assist NSCs therapy for the treatment of neurodegenerative diseases.

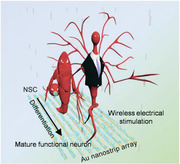

## 1 Introduction

Neurodegenerative diseases are global health threats that already affect the living quality of 50 million people worldwide, and this crisis is poised to continue to intensify.^[^ ^1^ ^]^ There is still no effective therapy that can halt or reverse neurodegeneration. Currently available medication only transiently delays the progression of brain injury in neurodegenerative diseases.^[^ ^2^ ^]^ Neural stem cells (NSCs) hold great promise for the treatment of neurodegenerative diseases by replacing damaged neurons following injury or disease to reconstruct neural circuits and restore lost neuronal functions.^[^ ^3^ ^]^ For NSC‐based therapy, the key to success is the efficient and rapid differentiation of NSCs into functional neurons.^[^ ^4^ ^]^ Electrical stimulation is one of the most effective and widely used modalities for promoting neural differentiation and augmenting neurogenesis by activating voltage‐gated ion channels.^[^ ^5^ ^]^ In addition, electrical stimulation has also shown remarkable results in synaptogenesis,^[^ ^6^ ^]^ neuron recruitment,^[^ ^7^ ^]^ and neuron survival.^[^ ^8^ ^]^ However, traditional electrical stimulation, in addition to surgically implanted electrodes, includes wires connected to the external power supply, and an additional surgery is required to remove the electrodes or wires after the stimulation is over.^[^ ^9^ ^]^ These procedures can result in secondary injuries and infections.^[^ ^10^ ^]^

Most recently, implantable devices capable of performing wireless electrical stimulation have received considerable attention for the treatment of neurodegenerative diseases because their use does not require an additional surgery, which minimizes the risk of infection.^[^ ^11^ ^]^ For example, Wang et al. developed a self‐electrified device composed of biocompatible galvanic cells on an ultraminiaturized conduit to offer electrical cues for peripheral nerve regeneration.^[^ ^12^ ^]^ In another study, Koo et al. reported a wireless, programmable electrical stimulator using biocompatible circuit elements and substrates to convert radio frequency power into electrical stimulation to facilitate peripheral nerve repair.^[^ ^13^ ^]^ Although these devices are successful at different levels in extending the existing damaged neurites or axons in sciatic nerve repair, their fabrication is either complex or the generated electrical signal is uncontrollable. In addition, the efficiency of these devices in improving the neuronal differentiation of NSCs and in NSC‐based therapy has not been demonstrated.

Herein, we report an implantable, wireless device made of Au nanostrip array for electrical stimulation of NSCs. The device promotes the rapid differentiation of NSCs to mature, functional neurons. We focused on the Au nanostrip array because Au is biocompatible and bioinert. Furthermore, the Au nanostrip array can be prepared on different substrates (e.g., Si or polyimide (PI)) via facile microfabrication. When placed in a changing magnetic field provided by a rotating magnet, wireless electrical signals are generated on the Au nanostrips caused by the electromagnetic induction effect. The intensity of the generated electrical signals depends on the rotation speed and magnetic field strength. The present findings demonstrate that the Au nanostrip array‐based wireless device can stimulate NSCs to rapidly differentiate into mature functional neurons within 5 days. RNA sequencing (RNA‐Seq) revealed that the Au nanostrip‐based wireless electrical stimulation accelerated neuronal differentiation by activating voltage‐gated ion channels and triggering downstream signaling pathways. Importantly, the Au nanostrip array‐mediated wireless electrical stimulation also accelerated neuronal differentiation of NSCs in vivo in mice, with no occurrence of epilepsy, electric shock injuries, or other adverse effects. These results confirm the practicability of this promising strategy to functionalize the lining of implanted medical devices for the treatment of neurodegenerative diseases and nerve injuries.

## 2 Results and Discussion

### 2.1 Generation and Characterization of Electrical Signals

**Figure 1:**
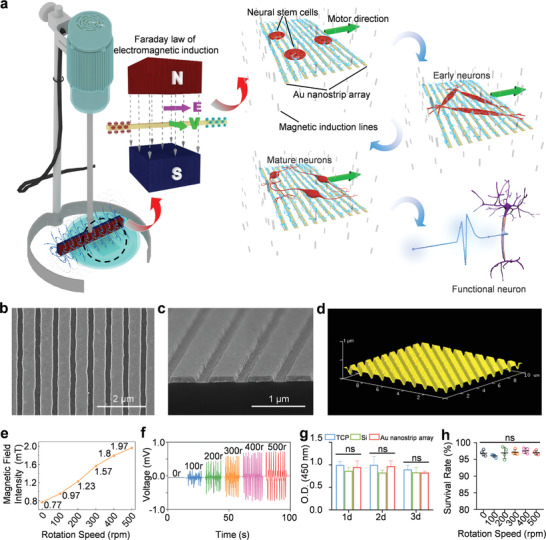
Wireless device for electrical stimulation of NSCs. a) Schematic illustration of the generation of electrical signals on the Au nanostrip array for stimulating the differentiation of NSCs into mature, functional neurons. Top b) and side view (c) SEM images of the Au nanostrip array. d) AFM image of the Au nanostrip array. e) Magnetic field intensity at the specific position (20 mm below the magnet) of the rotating magnetic field at different speeds (0, 100, 200, 300, 400, and 500 rpm). f) Generated induced voltages on the Au nanostrip array upon rotation of the magnet at 0, 100, 200, 300, 400, and 500 rpm. g) Viability of NSCs after culture on the TCP, Si, and Au nanostrip array for 1, 2, or 3 days. h) Survival rate of NSCs after culture on the Au nanostrip array for 3 days with the magnet rotating at 0, 100, 200, 300, 400, and 500 rpm. In panels g) and h), data are presented as the mean ± standard deviation (*n* = 4). ^ns^ *p* > 0.05.

A schematic illustration of the generation of electric signals from the Au nanostrip array‐based wireless device and its application in promoting the differentiation of NSCs into mature, functional neurons is shown in **Figure** **1**a. To ensure the generation of electrical signals, Au nanostrip arrays prepared on different substrates (e.g., Si or PI) were placed under a magnet, which was rotated using a motor (Figure S1 and Movie S1, Supporting Information). Based on the electromagnetic induction effect, the electrons in each Au nanostrip were driven from one side to the other when the nanostrip moved relative to the magnetic field. In our system, electric potential was generated in each nanostrip when the magnet rotated above the Au nanostrip array (Figure S2, Supporting Information). This generated electrical signal‐stimulated neuronal differentiation of NSCs (Movie S2, Supporting Information).

The Au nanostrip array was prepared through a facile nanoimprint technique by templating the nanostructured polycarbonate (PC) layer of a digital video (DVD) (Figure S3a–c, Supporting Information). Figure 1b–d shows scanning electron microscopy (SEM) and atomic force microscopy (AFM) images of the as‐obtained Au nanostrip array on the Si substrate. Each strip was ≈550 nm in width, and the gap between two adjacent strips was ≈200 nm. The water contact angle of the Au nanostrip array was < 90°, suggesting that the substrate was relatively hydrophilic (Figure S4, Supporting Information). The hydrophilic surface facilitates easy adhesion of cells.^[^ ^14^ ^]^ According to the law of electromagnetic induction, an induced potential is generated in each Au nanostrip when the substrate is placed in a rotating magnetic field, and the value of this potential depends on the intensity of the magnetic field and rotation speed of the magnet. We used a neodymium‐iron‐boron (NdFeB) magnet to generate the magnetic field. The magnet was placed 20 mm above the culture plate. At this distance, the static magnet generated a magnetic field with a density of 0.77 mT (Figure 1e). The magnetic field intensity increased to 0.97, 1.23, 1.57, 1.8, and 1.97 mT when the rotation speed of the magnet was increased to 100, 200, 300, 400, and 500 rpm, respectively. Figure 1f shows the voltage generated between the two ends of the Au nanostrip array when the magnet was rotated at different speeds. The maximum voltages generated on the 10 × 10 mm^2^ Au nanostrip array at 0, 100, 200, 300, 400, and 500 rpm rotating magnetic fields were 0, 0.17, 0.43, 0.59, 0.71, or 0.72 mV, respectively. The maximum induced output currents of the Au nanostrip array along the nanostrip direction at 0, 100, 200, 300, 400, and 500 rpm rotating magnetic field were 0, 10.5, 15.7, 21.8, 26.6, or 29.2 µA, respectively (Figure S5a,b, Supporting Information). Notably, in the presence of the rotating magnetic field, the output voltage generated on the Si substrate without the Au nanostrips was the same as that on the Au nanostrip array (Figure S5c, Supporting Information). However, the maximum output current on the Si substrate (0.038 µA) was 570 times lower than that generated on the Au nanostrip array (21.8 µA) (Figure S5d, Supporting Information). These findings suggest the critical role of the conductive Au nanostrips in generating efficient electrical stimulation in this wireless device. Furthermore, the generated electrical signals on the Au nanostrip array‐based wireless device had a typical sinusoidal pulse (Figure S6, Supporting Information), which has been demonstrated to be an effective stimulation for promoting neuronal differentiation of NSCs.^[^ ^12^, ^15^ ^]^ The pulse signals were attributed to periodic position changes between the rotating magnetic field and the Au nanostrip array, as illustrated in Movie S3 (Supporting Information).

NSCs isolated from the forebrains of mouse embryos were used as model cells to evaluate the cytocompatibility of the Au nanostrip array (Figure S7, Supporting Information). The Cell Counting Kit‐8 (CCK‐8) assay was performed to characterize the viability of NSCs cultured on tissue culture plates (TCPs), Si, or Au nanostrip arrays for 1, 2, or 3 days. The Au nanostrip array‐based device had good cytocompatibility, similar to that of the commercial culture plate (Figure 1g). This good cytocompatibility of the Au nanostrip array was further verified by live/dead staining results (Figure S8a,b, Supporting Information). We then evaluated the influence of the rotating magnetic field on the viability of NSCs by placing the Au nanostrip substrate under the magnet at different rotating speeds (0, 100, 200, 300, 400, and 500 rpm). Both CCK‐8 and live/dead staining results demonstrated > 96% viability of all NSC populations after 3 days of culture on the Au nanostrip array substrate (Figure 1h; and Figures S9 and S10, Supporting Information). These collective results demonstrate the good biocompatibility of the Au nanostrip array with NSCs and the lack of a negative influence of the rotating magnetic field on cell viability.

### 2.2 Electrical Stimulation Accelerates Differentiation of NSCs at the mRNA Level

**Figure 2:**
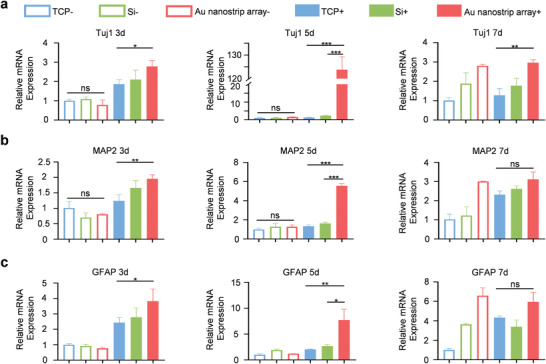
RT‐qPCR analysis of the expression of neural‐related genes a) *Tuj1*, b) *MAP2*, and c) *GFAP* for NSCs seeded on TCP, Si, or the Au nanostrip array and cultured without or with rotating magnetic field (300 rpm) for 3, 5, or 7 days. All data are presented as the mean ± standard deviation (*n* = 3). ^ns^ *p* > 0.05, *p < 0.05, **p < 0.01, ***p < 0.001.

To evaluate the influence of the electrical stimulation generated by the Au nanostrip‐based wireless device on the neuronal differentiation of NCSs, a real‐time quantitative polymerase chain reaction (RT‐qPCR) assay was performed under rotating magnetic field stimulation (300 rpm). Neuron‐specific class III beta‐tubulin (*Tuj1*), microtubule‐associated protein‐2 (*MAP2*), and glial fibrillary acidic protein (*GFAP*) were examined as representative genes to evaluate the neuronal differentiation of NSCs. Tuj1 is a tubulin involved in neurite outgrowth of neurons, which is present in immature neuronal cell bodies, dendrites, axons, and axon ends, and is considered a marker of early neuronal differentiation.^[^ ^16^ ^]^ MAP2 is critical for the morphological and functional differentiation of neurons, and is a typical hallmark of mature neurons.^[^ ^17^ ^]^ GFAP is mainly distributed in astrocytes of the central nervous system and is a typical marker of astrocyte activation.^[^ ^18^ ^]^ As shown in **Figure** **2**a,b, the expression of *Tuj1* and *MAP2* mRNAs of NSCs on different substrates (TCP‐, Si‐, and Au nanostrip array‐) in the absence of the rotating magnetic field was not significantly different after culture for 3 days, indicating that the substrates had no influence on the neuronal differentiation of NSCs. In sharp contrast, under a rotating magnetic field (300 rpm), expression of *Tuj1* and *MAP2* mRNAs of NSCs on the Au nanostrip substrate (Au nanostrip array+) was upregulated by 1.5‐ and 1.6‐fold, respectively, compared to the TCP+ group. The findings suggest that the electrical signals generated by the Au nanostrip array could promote neuronal differentiation of NCSs. After 5 days of culture, the NSCs in the TCP‐, Si‐, and Au nanostrip array groups still showed no significant difference in terms of the expression levels of *Tuj1* and *MAP2* mRNA. The mRNA expression of *Tuj1* and *MAP2* in the Au nanostrip array+ group was 97.6 and 4.2 times higher, respectively, than that in the TCP+ group, and 53.8 and 3.4 times higher, respectively, than that in the Si+ group. High expression levels of *Tuj1* and *MAP2* mRNAs in the Au nanostrip array+ group confirmed that the electrical stimulation generated by the Au nanostrip‐based wireless device accelerated the neuronal differentiation of NSCs. At day 7, the mRNA expression of *Tuj1* in the Au nanostrip array+ was 1.3 times higher than that in the TCP+ group, whereas the expression of *MAP2* mRNA in NSCs on different substrates with rotating magnetic field was not significantly different, probably because the NSCs in all groups had differentiated and tended to form mature neurons.

In addition, the trend of GFAP expression was similar to that of MAP2 expression, and the GFAP expression in the Au nanostrip array+ group was 3.8 and 2.9 times higher than that in the TCP+ and Si+ groups on day 5, respectively (Figure 2c). These results demonstrate that the electrical stimulation generated by the Au nanostrip‐based wireless device can also accelerate the differentiation of astrocytes, which can support the survival of neurons differentiated from NSCs.^[^ ^19^ ^]^

### 2.3 Electrical Stimulation Accelerates Differentiation of NSCs at the Protein Level

**Figure 3:**
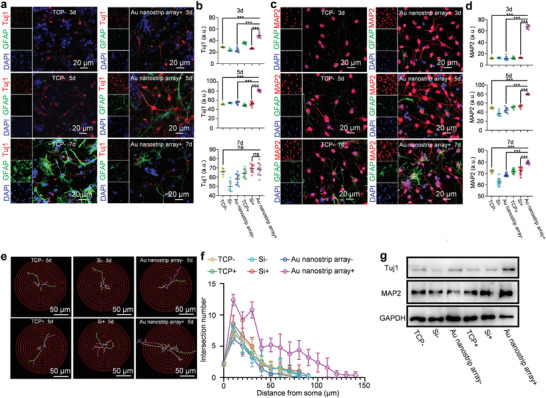
Au nanostrip array‐based wireless device accelerates neuronal differentiation of NSCs at the protein level. a) Confocal microscopy images of NSCs seeded on TCP or Au nanostrip array and cultured without or with the rotating magnetic field (300 rpm) for 3, 5, or 7 days. Tuj1 and GFAP were stained red and green, respectively. Cell nuclei were stained blue with 4',6‐diamidino‐2‐phenylindole (DAPI). b) Quantitative mean immunofluorescence intensity of Tuj1. c) Confocal microscopy images of NSCs seeded on TCP or Au nanostrip array and cultured without or with the rotating magnetic field (300 rpm) for 3, 5, or 7 days. MAP2 and GFAP were stained red and green, respectively. Cell nuclei were stained blue with DAPI. d) Quantitative mean immunofluorescence intensity of MAP2. b,d) At least 30 cells were analyzed in each group using Image J software. The data are presented as mean ± standard deviation; ^ns^ *p* > 0.05, ***p < 0.001. e) Images of 2D‐reconstructed neuronal morphologies that were traced and visualized based on the Tuj1 protein expression of NSCs seeded on TCP, Si, or Au nanostrip array, and cultured without or with the rotating magnetic field (300 rpm) on day 5. f) Sholl analysis of the neurite complexity of the 2D‐reconstructed neuronal morphologies presented in e). Neurites of ten neurons on each group were randomly measured. g) Western blot analysis of the Tuj1 and MAP2 protein expression of NSCs seeded on TCP, Si, or Au nanostrip array, and cultured without or with the rotating magnetic field (300 rpm) on day 5. Glyceraldehyde 3‐phosphate dehydrogenase (*GAPDH*) was used as the housekeeping gene.

To further evaluate the neuronal differentiation of NSCs, immunostaining was performed to detect the expression of the neuron‐specific protein markers Tuj1 and MAP2 (**Figure** **3**a–d; and Figures S11 and S12, Supporting Information). Confocal microscopy images and Tuj1 and MAP2 fluorescence intensity statistical analysis indicated that more cells expressed Tuj1 and MAP2 in the Au nanostrip array+ group than that in the TCP‐, Au nanostrip array‐, and Si+ groups after culture for 3 days. Furthermore, more typical neuronal morphologies, longer axons, and stronger expression of Tuj1 and MAP2 were observed in NSCs of the Au nanostrip array+ group than that in the TCP‐, Au nanostrip array‐, and Si+ groups at 5 days. No obvious differences in Tuj1 expression were found in NSCs among all groups on day 7. As shown in Figure S13 (Supporting Information), there were more proportion of MAP2 positive cells in the Au nanostrip array+ group (≈65%) compared with that in other groups (TCP‐, Au nanostrip array‐, and Si+ group) at day 5. There was no difference in the proportion of MAP2 positive cells in all the groups at day 7. Interesting, the proportion of MAP2 positive cells in the Au nanostrip array+ group at day 5 (≈65%) was close to that in other groups at day 7 (≈67%). These results indicated that the Au nanostrip‐based wireless electrical stimulation will accelerate neuronal differentiation without affecting the proportion of neurons that eventually differentiate. Furthermore, there was no difference in the proportion of GFAP positive cells in all the groups at day 5 or 7.

Based on the expression of Tuj1 on day 5 (Figure S11, Supporting Information), the neuronal morphologies of the differentiated NSCs in all groups were reconstructed by Sholl analysis (Figure 3e,f; and Figure S14, Supporting Information). The intersections of the concentric circles around the neuronal cell body (step = 10 µm) and the neurites of reconstructed neuronal morphology reflected the synaptic complexity and further revealed the development of neurons.^[^ ^20^ ^]^ No clear differences were observed among the TCP‐, Si‐, and Au nanostrip array‐ groups (axon length ≈100 µm) (Figure S14, Supporting Information). In contrast, the differentiated NSCs in the Au nanostrip array+ group exhibited multipolar characteristics of longer axons (≈230 µm) and increased neurite arborization than those in other groups (Figure S14, Supporting Information). These findings suggest that the Au nanostrip‐based wireless device could effectively promote the development of neurites, which strengthens the connections between neurons and lays the foundation for further neuronal functional interactions.

Western blot analysis further confirmed that the relative protein expressions of Tuj1 and MAP2 were the highest in the Au nanostrip array+ group among all groups at 5 days (Figure 3g; and Figure S15, Supporting Information), which was consistent with the RT‐qPCR and immunostaining results. The remarkable upregulation of the expression of the protein markers Tuj1 and MAP2 demonstrated that electrical stimulation not only facilitated neuronal differentiation at an early stage, but also promoted the differentiation of NSCs into mature neurons, and might even execute neuronal function, which is key for the treatment of neurodegenerative diseases by NSC‐based therapy.

### 2.4 Expression of Mature Functional Neurons

**Figure 4:**
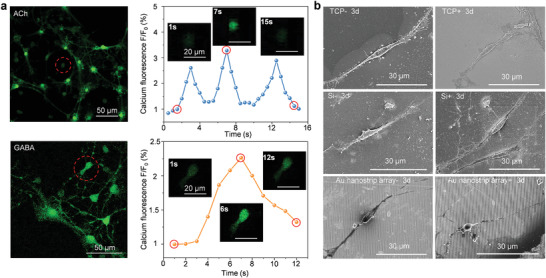
Evaluation of the functional neurons. a) Calcium dynamics within the differentiated neurons seeded on Au nanostrip array and cultured with rotating magnetic field (300 rpm) for 5 days (calcium ion indicated in green). Left panels show images of neurons after calcium dye (Fluo‐4 AM) loading. Right panels show time‐series imaging of relative fluorescence intensity change (%ΔF/F) for individual neurons after stimulation by acetylcholine (ACh) or g‐aminobutyric acid (GABA) neurotransmitters. The inset shows the fluorescence intensity expression of the cell body in the selected region at different time points. b) SEM images of the differentiated neurons on TCP, Si, or Au nanostrip array, cultured without or with rotating magnetic field (300 rpm) for 3 days.

We examined the neuronal function of differentiated NSCs using fluorescence imaging of calcium ion (Ca^2+^) dynamics. One typical function of mature neurons is that they can respond to neurotransmitters for transmitting signals.^[^ ^21^ ^]^ The presence of certain neurotransmitters generally causes transmembrane ion (e.g., Ca^2+^) flow in neurons.^[^ ^22^ ^]^ To this end, Ca^2+^ spark experiments can be used to identify the function of mature neurons. In the present study, we used two neurotransmitters, acetylcholine (ACh) and g‐aminobutyric acid (GABA), to evaluate the function of differentiated NSCs obtained under the Au nanostrip array‐mediated wireless electrical stimulation for 5 days (**Figure** **4**a). After the addition of ACh, the fluorescence intensity (indicating the intracellular concentration of Ca^2+^) of the differentiated NSCs increased rapidly and reached a peak value at 3 s before rapidly decreasing. This process was repeated three times until the cells reached a resting state at 15 s (Movie S4, Supporting Information). Similar fluorescence changes, i.e., increase and then decrease, were observed when GABA was introduced into the differentiated NSCs (Movie S5, Supporting Information). More importantly, the fluorescence intensity changed along the axons, indicating that the differentiated NSCs had the typical function of mature neurons in transmitting signals. In contrast, the dopamine (DA) and l‐glutamate (Gln) neurotransmitters did not lead to changes in the concentration of intracellular Ca^2+^ (Figure S16, Supporting Information). These collective results indicate that the mature neurons formed on the Au nanostrip substrate with wireless electric stimulation were AChergic or GABAergic neurons. The differentiated NSCs cultured on TCP did not show changes in the fluorescence intensity of imaging Ca^2+^ dynamics after introducing the ACh or GABA neurotransmitters until day 10 (Figure S17 and Movies S6 and S7, Supporting Information). Hence, the Au nanostrip‐based wireless device could accelerate the differentiation of NSCs into functional neurons at least 5 days earlier than that observed in the cells without treatment.

SEM images presented in Figure 4b show that the NSCs in the Au nanostrip array+ group exhibited a rounder cell body with longer axons at day 3 compared to NSCs on other substrates. The neurites of the differentiated NSCs in the Au nanostrip array+ group were further extended to hundreds of micrometers on day 5 (Figure S18, Supporting Information). These results suggest that the Au nanostrip‐based wireless device could accelerate the differentiation of NSCs into mature neurons. For the treatment of neurodegenerative diseases, it is important for NSCs to differentiate into mature and functional neurons. Generation of neurites as antennae that receive neurotransmitter stimulation are representative features of mature and functional neurons.^[^ ^23^ ^]^

### 2.5 Mechanism of Electrical Stimulation Accelerates Differentiation of NSCs

Voltage‐gated ion channels are generally involved in electrical stimulation‐induced neuronal differentiation of NSCs. To check whether the wireless device promoted neuronal differentiation through the voltage‐gated ion channel, RNA‐seq was performed for NSCs after culture in TCP‐ and Au nanostrip array+ groups for 48 h. Compared with the TCP‐ group, 131 genes were upregulated and 176 genes were downregulated in the Au nanostrip array+ group. The significantly differentially expressed genes for NSCs among the various groups are presented in the heat map shown in Figure S19a (Supporting Information).

**Figure 5:**
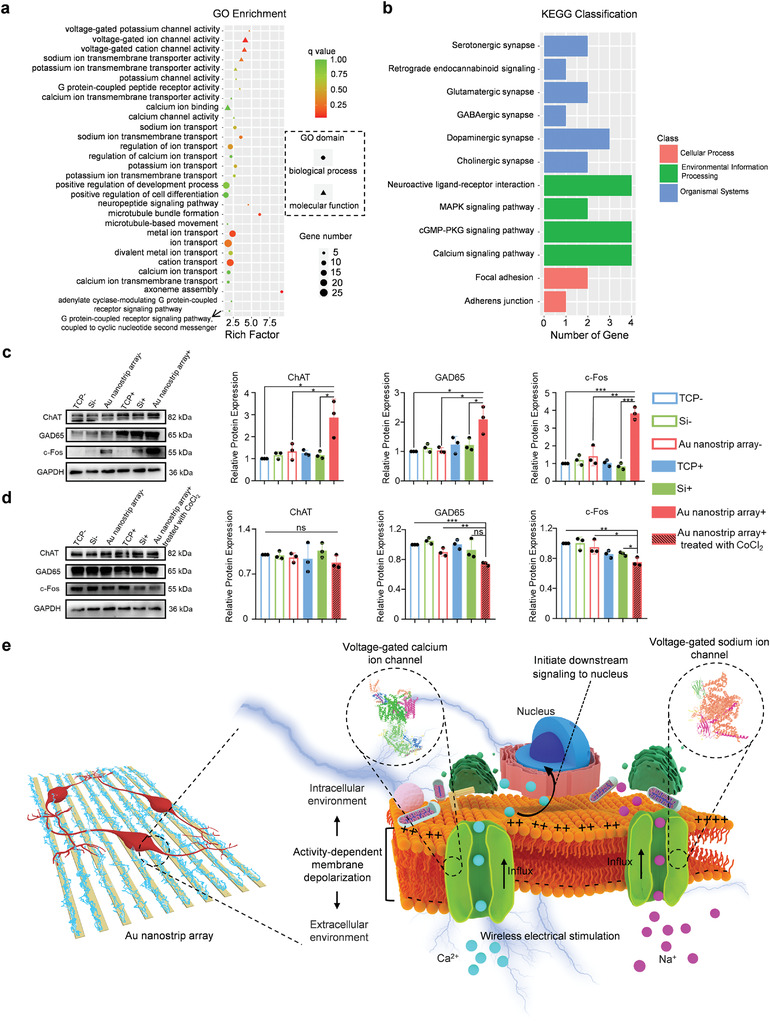
Mechanism underlying NSC differentiation promoted by the Au nanostrip‐based wireless device. a) GO functional enrichment analysis of differentially expressed genes between the Au nanostrip array+ and TCP‐ groups. b) KEGG pathway classification of differentially expressed genes between the Au nanostrip array+ and TCP‐ groups. c) Western blot analysis of the ChAT, GAD65, and c‐Fos protein expression of NSCs seeded on TCP, Si, or Au nanostrip array and cultured without or with rotating magnetic field (300 rpm) on day 5. Glyceraldehyde 3‐phosphate dehydrogenase (GAPDH) was used as the housekeeping gene. Quantitative analysis data obtained using Image J software are presented as mean ± standard deviation (*n* = 4); ^ns^ *p* > 0.05, *p < 0.05, **p < 0.01, ***p < 0.001. d) Western blot analysis of ChAT, GAD65, and c‐Fos protein expressions by NSCs seeded on TCP, Si, or Au nanostrip array and cultured without or with rotating magnetic field (300 rpm) on day 5. The NSCs in the Au nanostrip array+ group were treated with 3 × 10^−3^ m CoCl_2_ before the rotating magnetic field was applied. GAPDH was used as the housekeeping gene. Quantitative analysis data acquired by Image J software are presented as mean ± standard deviation (*n* = 4); ^ns^ *p* > 0.05, *p < 0.05, **p < 0.01, ***p < 0.001. e) Diagram of the mechanism of NSCs differentiation promoted by the Au nanostrip‐based wireless device.

Next, gene ontology (GO) enrichment analysis was performed to elucidate the functional properties of the significantly differentially expressed genes when NSCs were cultured on Au nanostrip substrates with a rotating magnetic field (300 rpm) compared with those cultured on the TCP (**Figure** **5**a; and Figure S19b,c, Supporting Information). Some significantly enriched GO terms related to molecular function and biological processes were associated with neuronal differentiation, such as positive regulation of development process, central nervous system development, positive regulation of cell differentiation, microtubule bundle formation, dendrites, axons, and G protein‐coupled peptide receptor activity. Notably, many of the enriched GO terms were related to voltage‐gated ion channel activity and the process of ion transmembrane transport; these included voltage‐gated potassium channel activity, voltage‐gated ion channel activity, voltage‐gated cation channel activity, sodium ion transmembrane transporter activity, ion channel binding, Ca^2+^ channel activity, regulation of Ca^2+^ ion transport, sodium ion (Na^+^) transmembrane transport, and mitogen‐activated protein kinase (MAPK) signaling pathway. These results implied that voltage‐gated ion channels may participate in the process by which Au nanostrip‐based wireless devices accelerate neuronal differentiation of NSCs.

To further link basic genomic information to higher‐order biological functions, Kyoto Encyclopedia of Genes and Genomes (KEGG) analysis was performed to examine the signaling pathways that were significantly enriched in differentially expressed genes between the TCP‐ and Au nanostrip array+ groups (Figure 5b). Among these pathways, glutamatergic synapse, dopaminergic synapse, cholinergic synapse, serotonergic synapse, and neuroactive ligand–receptor interactions were strongly associated with neuronal development and functional establishment. Additionally, the typical enriched terms in cellular processes, such as focal adhesion and adherens junctions, are related to the neuronal differentiation process. Notably, the MAPK, cGMP‐protein kinase G (PKG), and Ca^2+^ signaling pathways were all enriched in voltage‐gated ion channel activity. The KEGG analysis revealed that Au nanostrip array‐mediated wireless electrical stimulation could effectively promote the neuronal differentiation of NSCs by activating voltage‐gated ion channel activity‐related signaling pathways.

To further determine the more effective facilitation of wireless electrical stimulation generated on the Au nanostrip array in promoting neuronal differentiation of NSCs compared with that on the Si substrate under the same conditions, the gene expression profile of NSCs in the Si+ group was further analyzed by RNA sequencing and compared with that in Au nanostrip array+ groups (Figure S20a, Supporting Information). GO analysis indicated that most of the upregulated genes in the Au nanostrip array+ group were significantly enriched in terms associated with neuronal differentiation (including positive regulation of cell differentiation, neuron differentiation, neurogenesis, and neuron development, etc.) or voltage‐gated ion channel activity (including voltage‐gated ion channel activity, voltage‐gated cation channel activity, and Ca^2+^ ion transmembrane transport, etc.) (Figure S20b,c, Supporting Information). KEGG analysis further confirmed that the typically enriched pathways were related to the neuronal differentiation process or voltage‐gated ion channel activity (Figure S20d, Supporting Information). The above results demonstrated that compared to electrical signals generated by a Si substrate under a rotating magnetic field (300 rpm), electrical stimulation by the Au nanostrip‐based wireless device could activate voltage‐gated ion channels, trigger downstream signaling pathways, and thus promote the neuronal differentiation of NSCs more efficiently.

The RNA‐seq results prompted western blot analysis to confirm that the relative protein expression of choline acetyltransferase (ChAT), a AChergic neuron‐specific marker,^[^ ^24^ ^]^ GAD65, a GABAergic neuron‐specific marker,^[^ ^25^ ^]^ and c‐Fos, a reporter gene for neuronal activation by Ca^2+^ influx,^[^ ^5b^ ^]^ in the NSCs of all groups at 5 days (Figure 5c). The relative protein expression of ChAT, GAD65, and c‐Fos in the Au nanostrip array+ group was the highest than that in other groups. In addition, the marked increased protein expression of ChAT, GAD65, and c‐Fos disappeared when NSCs in Au nanostrip array+ group were treated with 3 × 10^−3^ m CoCl_2_, a nonspecific voltage‐gated Ca^2+^ channel blocker,^[^ ^26^ ^]^ before the rotating magnetic field was applied (Figure 5d). The relative protein expression of Ca^2^⁺/calmodulin‐dependent protein kinase II (CaMKII), a central coordinator and executor of Ca^2+^ signal transduction,^[^ ^27^ ^]^ and phosphorylated CaMKII (p‐CaMKII), the activated form of CaMKII, in NSCs cultured on Au nanostrip array and either untreated or treated with 3 × 10^−3^ m CoCl_2_ before the rotating magnetic field was applied was further analyzed on day 5 (Figure S21, Supporting Information). The wireless electrical stimulation generated on the Au nanostrip array did not significantly affect the total amount of CaMKII compared to that in TCP‐group within 5 days. However, the p‐CaMKII was remarkably upregulated, indicating the effective activation of CaMKII. This upregulation of p‐CaMKII in NSCs of the Au nanostrip array+ group was largely inhibited by CoCl_2_. The collective results demonstrate that the wireless electrical stimulation generated on the Au nanostrip array can effectively promote functional neuronal differentiation through voltage‐gated Ca^2+^ channels and Ca^2+^ signaling pathway, consistent with the RNA‐seq results.

As illustrated in Figure 5e, wireless electrical stimulation effectively triggered Ca^2+^ and Na^+^ influx through the voltage‐gated ion channels. The voltage‐gated ion channels are the key transducers of cell surface membrane potential, which cause activity‐dependent membrane depolarization and initiate downstream signaling associated with neural differentiation to the nucleus.^[^ ^28^ ^]^ In contrast, as electrophysiological switches of the mature neuron in signal transduction of the nervous system, the expression of neuronal voltage‐gated Na^+^ and Ca^2+^ channels is an essential hallmark of neuronal differentiation, and is crucial for the generation of an action potential, suggesting that NSCs differentiate into fully functional neurons.^[^ ^23^, ^29^ ^]^ There is a positive feedback loop between NSC differentiation and voltage‐gated ion channel activity. Differentiated neurons gradually activate voltage‐gated ion channels to express electroactivity. The electroactivity further promotes regulation of the intrinsic excitability of cells to accelerate the developmental program,^[^ ^28^, ^29^ ^]^ which is pivotal in the establishment of synaptic connections between newly generated neurons and pre‐existing neurons to form functional integrated neural circuits.^[^ ^28^, ^30^ ^]^ Wireless electrical stimulation reinforces this positive feedback loop through human factors.

NSCs in the Au nanostrip array+ group also differentiated faster than those seeded on a flat Au film under a rotating magnetic field (Figure S22a–c, Supporting Information). The accelerated differentiation of NSCs may be because of the effective electrical stimulation caused by the micro‐ or nanostructure of the Au nanostrip array. During electrical stimulation, the charge accumulated on both edges of the conductor as the stimulus sites. The construction of nanostrips by the nanoimprint technique substantially increased the number of edges per unit area, thus increasing the number of stimulation sites and assisting faster differentiation and development of NSCs.

### 2.6 In Vivo Study of Accelerated Neuronal Differentiation of NSCs

We further evaluated the performance of the Au nanostrip array‐based wireless device in promoting neuronal differentiation of NSCs in vivo. To better fit the neural tissues, an Au nanostrip array was fabricated on biocompatible and flexible PI using the same nanoimprint technology (Figure S23a–d, Supporting Information).^[^ ^31^ ^]^ The PI substrate minimized biological incompatibility, as well as decreased the physical and mechanical mismatch between the implantable devices and neural tissues.^[^ ^32^ ^]^ The Au nanostrip array on PI generated similar induced output electrical signals under a rotating magnetic field to that on the Si substrate (Figure S24, Supporting Information). NSCs also exhibited good viability. The main extensional neurites could anchor to the substrate surface, indicating the good biocompatibility of the Au nanostrips on PI (Figure S25a–c, Supporting Information).

**Figure 6:**
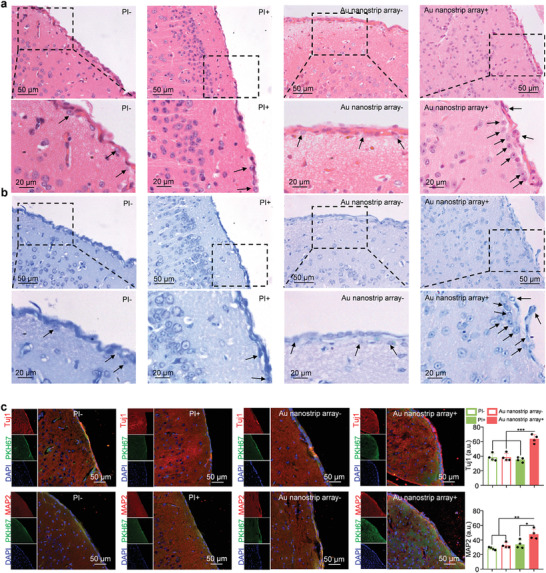
Au nanostrip array‐mediated electrical stimulation promotes neuronal differentiation of NSCs in vivo. a) H&E staining and b) Nissl staining results in the cerebral cortex section obtained at the interface of substrates (PI or Au nanostrip array) seeded with NSCs and without or with rotating magnetic field (300 rpm) on day 7. Black arrows point to neuron‐like cells. c) Immunofluorescent images of the cerebral cortex section obtained at the interface of substrates (PI or Au nanostrip array) seeded with NSCs and without or with rotating magnetic field (300 rpm) on day 7. In the upper row of panels, Tuj1 and cell membrane were stained red and green, respectively. Nuclei of cells were stained blue by DAPI. In the lower row of panels, MAP2 and cell membrane were stained red and green, respectively. Nuclei of cells were stained blue by DAPI. Quantitative mean immunofluorescence intensity of Tuj1 or MAP2 in each group are presented as mean ± standard deviation (*n* = 4); ^ns^ *p* > 0.05, *p < 0.05, **p < 0.01, ***p < 0.001.

The Au nanostrip array on the PI was cut into round disks 5 mm in diameter. After seeding with NSCs, the Au nanostrip substrates were implanted in 5 mm skull holes and attached to the cerebral cortex of C57 mice (Figure S26a–g, Supporting Information). Half of the mice were then exposed to a rotating magnetic field (300 rpm) for 7 days. PI substrates seeded with NSCs in the absence of Au nanostrip arrays were also implanted in the same way and served as the control group. Hematoxylin and eosin (H&E) staining revealed that more neuron‐like cells were generated at the interfaces between the substrate and the cerebral cortex in the Au nanostrip array+ group than in the Au nanostrip array‐, PI‐, and PI+ groups (**Figure** **6**a; and Figure S27, Supporting Information). The Nissl staining results (Figure 6b) further confirmed that neurons were formed in the Au nanostrip array+ group, suggesting that Au nanostrip array‐mediated wireless electrical stimulation could effectively promote the differentiation of NSCs into mature neurons in vivo.

To confirm that the neurons observed on the surface of the cortex were differentiated from the implanted NSCs, the NSCs seeded on the different substrates were pre‐labeled with PKH67 (Figure S28, Supporting Information) before implantation. Figure 6c shows representative fluorescence images of the obtained cortex together with different implanted devices, in which Tuj1 and MAP2 were stained red. For the Au nanostrip array group under a rotating magnetic field (300 rpm), a large number of neural‐specific marker‐positive cells were found around the implant. The overlapping of PKH67 and Tuj1 (or MAP2) confirmed that the generated neurons were differentiated from the implanted NSCs. These results demonstrate that Au nanostrip array‐mediated wireless electrical stimulation could accelerate the differentiation of NSCs into mature neurons in vivo, consistent with the in vitro results. Notably, no obvious difference was observed among all groups when the tissues were stained to reveal GFAP (Figure S29, Supporting Information), suggesting that wireless electrical stimulation had no influence on the differentiation of NSCs to astrocytes in vivo. The resistivity of the brain tissue was ≈2.84 Ω m, which was too large for Au nanostrip array (2.4 × 10^−8^ Ω m). Therefore, induced current may only exist in the Au nanostrip array, or the induced current in brain tissue can be completely ignored. No epilepsy, electric shock injuries, or other adverse effects developed in any of the mice.

## 3 Conclusion

We developed a novel and versatile strategy for generating wireless electrical signals on an Au nanostrip array with a rotating magnetic field to accelerate neuronal differentiation of NSCs, and achieved mature functional neurons within 5 days. The Au nanostrip array‐based wireless device accelerated the neuronal differentiation of NSCs at the mRNA, protein, and physiological functional levels in vitro. Transcriptome sequencing analysis revealed that wireless electrical stimulation could effectively activate voltage‐gated ion channels, cause activity‐dependent membrane depolarization, and initiate the downstream signaling associated with neural differentiation. We also demonstrated that the Au nanostrip array‐mediated wireless electrical stimulation could accelerate the differentiation of NSCs into mature neurons in vivo. This work reveals a new route for accelerating the neuronal differentiation of NSCs, which will improve the curative effect of NSCs treatment of neurodegenerative diseases. It is a promising strategy for functionalizing the lining of implanted medical devices for neurodegenerative diseases and nerve injuries.

## 4 Experimental Section

### Preparation and Characterization of Au Nanostrip Arrays

Au nanostrip arrays were prepared by nanoimprint lithography (NIL) using a low‐cost DVD disc as a template. First, a polycarbonate layer was obtained by separating the DVD disc from the middle using a scalpel. The diluted polydimethylsiloxane (PDMS) (pre‐polymer:curing agent = 10:1) was coated on a polycarbonate layer under vacuum pumping conditions, and then cured at 70 °C for 2 h to prepare the PDMS nanoimprinting template. Then, LOL2000 (Shipley Company, Marlborough, MA), used as the bottom photoresist, was spin‐coated on a silicon substrate with a thickness of 300 nm and then cured at 150 °C for 3 min. The substrate with cured LOL was spin‐coated with AR‐N 4340 top photoresist (diluted 1:4 with AR 300‐12) at 6000 rpm. The PDMS nanoimprinting template was imprinted on a Si substrate coated with two layers of photoresist and kept for 30 min. The substrate covered with the PDMS template was heated at 110 °C for 150 s to soft‐bake the AR‐N 4340. This was followed by detachment of the PDMS mold from the substrate. After removing the PDMS nanoimprinting template, the photoresist coated on the Si substrate was exposed to ultraviolet light at an exposure dose of 9 mW cm^−2^ for 25 s. After crosslink baking at 120 °C for 2 min and development, the Au layer with a thickness of ≈100 nm was thermally evaporated. After lift‐off of the photoresist and cleaning with deionized water, the Au nanostrip array on the Si substrate was obtained. Au nanostrip arrays on PI (PI‐2611; DuPont, Wilmington, DE) substrate were prepared in the same way. Before conducting the NIL, PI was spin‐coated onto a cleaned silicon wafer, followed by a curing process. The morphology and structure were characterized by using SEM (S‐4800, Hitachi, Japan) and AFM (Icon, Bruker, USA). X‐ray diffraction patterns were recorded on a Bruker D8 Advance powder diffractometer equipped with a copper K*α* sealed tube.

### Generation of Wireless Electrical Stimulation on Au Nanostrip Array

The rotating magnet consisted of an NdFeB magnet and motor. The NdFeB magnet was installed at the end of the motor shaft and rotated with the shaft to generate a rotating magnetic field. The rotation speed of the magnet was adjusted using a motor controller. The prepared Au nanostrip array was placed into the cell culture plate. The culture plate was placed on the side near the motor shaft center under the NdFeB magnet. Considering the height of the cell culture plate, the distance from the NdFeB magnet to the Au nanostrip array under actual working conditions was 20 mm. A CH‐1800 gaussmeter (Reanow, China) was used to measure the magnetic field intensity at a specific position (20 mm below the magnet) with the NdFeB magnet at different rotational speeds (0, 100, 200, 300, 400, and 500 rpm). According to the law of electromagnetic induction, wireless electrical stimulation is generated on each Au nanostrip when the substrate is placed in a rotating magnetic field. A 2400 source measurement unit instrument (Keithley, USA) was used to measure the output voltages and currents of the wireless electrical stimulation generated on the Au nanostrip array when the magnet was rotated at 0, 100, 200, 300, 400, and 500 rpm by wire connection. The output voltages and currents of the wireless electrical stimulation generated on Si were measured in the same manner.

### NSC Extraction and Culture

NSCs were isolated from the forebrains of E13.5 C57 BL/6 mouse embryos obtained from Qilu Hospital of Shandong University. Animal care and experiments were performed in accordance with the protocols approved by the Ethics Committee of Shandong University, Qilu Hospital (DWLL‐2019‐24). NSCs were cultured at 5 × 10^5^ cells per 60 mm dish in a growth medium consisting of neurobasal medium supplemented with 2% B‐27, 1% penicillin/streptomycin, 1% glutaMAXTM‐1, basic fibroblast growth factor (bFGF, 20 ng mL^−1^), and epidermal growth factor (EGF, 20 ng mL^−1^) for proliferation. NSCs were maintained at 37 °C in a humidified atmosphere with 5% CO_2_. For differentiation of NSCs, the neurospheres were collected and dissociated into individual cells. The NSCs were seeded on different substrates (TCP, Si, or Au nanostrip array) that were precoated with laminin (1 µg mL^−1^) and cultured in neurobasal medium supplemented with 1% fetal bovine serum (FBS), 2% B‐27, 1% glutaMAXTM‐1, and 1% penicillin/streptomycin in 24‐well culture plates to allow adhesion. After 12 h, the medium was replaced with neuronal differentiation medium (neurobasal medium supplemented with 2% B‐27, 1% glutaMAXTM‐1, and 1% penicillin/streptomycin) without FBS for subsequent experiments. For experimental reliability, the third to fifth passage of NSCs was used for the experiments.

### Wireless Electrical Stimulation of NSCs

NSCs were seeded on different substrates (TCP, Si, or Au nanostrip array) at a density of 5 × 10^4^ cm^−2^. The different substrates were placed in the cell culture plate, which was placed on the side near the motor shaft center under the NdFeB magnet (20 mm). A rotating magnetic field (300 rpm) was applied to the different substrates when NSCs were stimulated. The daily stimulation duration was 20 min for 3, 5, or 7 days. Time‐matched control NSCs were kept without rotating magnetic field (300 rpm) excitation during the same period.

### Cytocompatibility of the Au Nanostrip Array

Different substrates (TCP, Si, or Au nanostrip array) were first sterilized by autoclaving. NSCs were seeded on different substrates in 24‐well culture plates at an initial density of 5 × 10^4^ cm^−2^ and incubated in neuronal differentiation medium (neurobasal medium, 2% B‐27, 1% glutaMAXTM‐1, and 1% penicillin/streptomycin). After 72 h of culture, the neuronal differentiation medium was changed to neurobasal medium containing 0.5 × 10^−6^ m calcein AM and 3 × 10^−6^ m PI. After incubation for 30 min at 37 °C, the cells were washed three times with PBS and observed by fluorescence microscopy using a IX73 microscope (Olympus, Japan). Image J software was used to count the number of living or dead cells and calculate the cell survival rates. Live/dead cell staining of NSCs after culture on the Au nanostrip array with magnet rotation at 0, 100, 200, 300, 400, and 500 rpm for 3 days was performed in the same way, except for a daily 20 min stimulation.

For further quantitative evaluation of cell viability, NSCs were cultured on different substrates (TCP, Si, or Au nanostrip arrays) in 24‐well culture plates at an initial density of 5 × 10^4^ cm^−2^ with neuronal differentiation medium for 1, 2, or 3 days (*n* = 3 per group). After the specified time period (1, 2, or 3 days), the culture medium was replaced with 500 µL serum‐free neurobasal medium containing 10% CCK‐8 solution. After incubation at 37 °C for 1 h, 100 µL of the medium was transferred from each well to a 96‐well culture plate. The level of production of water‐soluble formazan dye was assayed using a Multiscan MK3 microplate reader (Thermo Fisher Scientific, USA) at 450 nm. Viability of NSCs after culture on the Au nanostrip array with magnet rotation of 0, 100, 200, 300, 400, and 500 rpm for 1, 2, or 3 days was quantified in the same way, except for the daily 20 min stimulation.

### RT‐qPCR Assay

NSCs were cultured on different substrates (TCP, Si, or Au nanostrip array) with or without a rotating magnetic field (300 rpm) for 3, 5, or 7 days. Total RNA was extracted from the NSCs (5 × 10^5^ cells) in different groups using TRIzol Reagent (Invitrogen, USA). RNA concentration and purity were determined using a Q‐5000 spectrophotometer (Quawell, China) at 260/280 nm. RT‐qPCR analysis of one housekeeping gene (*β*‐actin) and the three target genes, *Tuj1*, *MAP2*, and *GFAP*, was performed using the Light Cycler 96 system (Roche Life Science, Switzerland). The relative transcript levels of the target gene were normalized to that of *β*‐actin and expressed as mean ± standard deviation (*n* = 3 per group). Primer information is provided in Table S1 (Supporting Information).

### Western Blot Analysis

Total proteins were extracted from the NSCs (1 × 10^6^ cells) on different substrates (TCP, Si, or Au nanostrip array) with or without a rotating magnetic field (300 rpm) at 5 days using RIPA lysis buffer (Beyotime Biotechnology, China). The BCA Protein Assay Kit (Beyotime Biotechnology) was used to quantify total protein concentrations. After dilution with 5× loading buffer, equal amounts of protein were resolved by sodium dodecyl sulfate–polyacrylamide gel electrophoresis. Proteins were electrophoresed initially at 110 V for 90 min or longer, and then transferred to polyvinylidene difluoride membranes at 300 mA for 1 h or longer using a wet transfer system. The membranes were blocked with 5% nonfat milk for 1 h at room temperature. Next, the membranes were incubated with primary antibodies against GAPDH (1:2000, Affinity, China), Tuj1 (1:2000, Abcam, UK), MAP2 (1:2000, Abcam), ChAT (1:2000, Abcam), GAD65 (1:2000, Proteintech, USA), c‐Fos (1:2000, Proteintech), CaMKII (1:2000, Proteintech), and p‐CaMKII (1:1000, Affinity) overnight at 4 °C. The membranes were washed with tris‐buffered saline containing with 0.1% Tween 20 detergent and then incubated with secondary antibodies at room temperature for 1 h. Finally, the chemiluminescent signal was developed using ECL kit reagents (Millipore, USA) and detected using X‐OMAT BT Film (Kodak, USA).

### Immunofluorescence Staining

NSCs were cultured on different substrates (TCP, Si, or Au nanostrip array) with or without a rotating magnetic field (300 rpm) for 3, 5, or 7 days. The cells were washed with PBS, fixed with 4% paraformaldehyde at room temperature for 15 min, permeabilized with 0.1% Triton X‐100 for 15 min, and blocked with 1% bovine serum albumin for 30 min at room temperature. After blocking, the cells were incubated overnight, at 4 °C, with the primary antibodies at 1:1000 dilution against Tuj1 (mouse monoclonal anti‐Tuj1, Abcam), 1:10 000 dilution against MAP2 (chicken polyclonal anti‐MAP2, Abcam), and 1:500 dilution against GFAP (rabbit polyclonal anti‐GFAP, Proteintech). Alexa Fluor 568 conjugated goat anti‐mouse, Alexa Fluor 594 conjugated goat anti‐chicken, and Alexa Fluor 488 conjugated goat anti‐rabbit secondary antibodies diluted 1:2000 in 3% bovine serum albumin were used to stain Tuj1, MAP2, and GFAP for 2 h at room temperature, respectively. After rinsing off the secondary antibody with PBS, the nuclei of cells were stained with 4',6‐diamidino‐2‐phenylindole (Life Technology, USA) at 1:5000 dilution for 3 min. The stained samples were observed and photographed using a LSM800 confocal laser scanning microscope (Carl Zeiss, Germany). The quantitative mean immunofluorescence intensity of neuronal marker expression in NSCs was further measured using Image J software. The 2D‐reconstructed neuronal morphology was performed using the Simple Neurite Tracer (from an open‐source project hosted on GitHub). Neurite complexity was further analyzed by Sholl analysis (from an open‐source project hosted on GitHub). Neurites from ten neurons in each group were randomly measured.

### Morphology of the Differentiated NSCs

SEM using a S‐4800 microscope (Hitachi) was used to visualize the differentiation and interactions of NSCs on different substrates. After culturing of NSCs on different substrates (TCP, Si, or Au nanostrip array) with or without a rotating magnetic field (300 rpm) for 3 or 5 days, cells were prepared for SEM by alcohol gradient dehydration. Cells were fixed with 2.5% glutaraldehyde solution for 30 min at room temperature. After washing with PBS three times, NSCs were further dehydrated using an alcohol gradient (30%, 50%, 70%, 80%, 90%, 95%, and 100%) and lyophilized at −60 °C for 6 h. Finally, the samples were coated with gold prior for SEM observation.

### Ca^2+^ Imaging and Analysis

The fluorescence imaging of the Ca^2+^ dynamics of NSCs cultured on Au nanostrip array with a rotating magnetic field (300 rpm) for 5 days was performed using a fluorescent Ca^2+^ indicator (Fluo‐4 AM, Beyotime Biotechnology). Briefly, neurons on different substrates were washed with PBS three times and incubated with 0.5 × 10^−6^ m Fluo‐4 AM for 10 min at 37 °C in HEPES (Sigma‐Aldrich, USA). After incubation with Fluo‐4 AM, the neurons were washed with PBS and incubated for another 20 min to allow complete de‐esterification of the dye. A LSM800 confocal laser scanning microscope (Carl Zeiss) was used to observe and photograph the neurons labeled with Fluo‐4 AM. Neurotransmitters (acetylcholine, g‐aminobutyric acid, dopamine, or glutamine) at a concentration of 500 × 10^−6^ m were used to stimulate neurons to trigger Ca^2+^ sparks. The relative fluorescence intensity change (%ΔF/F) for individual neurons showing the change in intracellular concentration of Ca^2+^ after stimulation by the neurotransmitters was calculated using MATLAB software (MathWorks, USA). The fluorescence imaging of the Ca^2+^ dynamics of the NSCs cultured on TCP without a rotating magnetic field (300 rpm) for 7, 9, or 10 days was performed in the same way.

### RNA‐seq and Bioinformatics

NSCs (3 × 10^6^ cells) in the TCP‐, Si+, and Au nanostrip array+ groups were treated with TRIzol Reagent (Invitrogen) at 6 h after the second stimulation with a rotating magnetic field (300 rpm). The samples were sent to the Shanghai Biotechnology Corporation for RNA‐seq analysis. Briefly, mRNAs were isolated from total RNA using the RNeasy Mini Kit (QIAGEN, USA). Subsequently, paired‐end libraries were synthesized using the TruSeq RNA Sample Preparation Kit (Illumina Inc., USA). Purified libraries were evaluated using a Qubit 2.0 Fluorometer (Life Technologies, USA) and a 2100 bioanalyzer (Agilent Technologies, USA) to confirm the insert size and calculate the mole concentration. Sequencing was performed on the Illumina HiSeq 2500 device. Subsequently, raw reads were preprocessed by removing rRNA reads, adapters, short‐fragment reads, and other low‐quality reads. The cleaned reads were aligned to the mouse reference genome to map out the reads with two mismatches using Tophat v2.0.9. After mapping, differentially expressed genes were analyzed using Cuffdiff. The differentially expressed genes were selected using the following filter criteria: false discovery rate ≤ 0.05 and fold‐change ≥ 2. Finally, the GO functional enrichment and KEGG pathways significantly affected in differentiated NSCs were detected by using Goatools v0.5.9 and KOBAS v2.0.

### Subcranial Implantation and Histological Analysis

To better fit the neural tissues, an Au nanostrip array was fabricated on a biocompatible and flexible PI substrate using the same nanoimprint technology. The PI and Au nanostrip arrays were cut into circles of 5 mm diameter using a punch. After autoclaving, the NSCs (5 × 10^5^ cells) labeled with PKH67 were seeded on different substrates (PI or Au nanostrip array) precoated with laminin (Sigma‐Aldrich). After 1 day, the PKH67 prelabeled NSC‐seeded substrates (PI or Au nanostrip array) were implanted under the skulls of 6‐week‐old C57 BL/6 mice (at least four mice per group). Briefly, the mice were anesthetized by an intravenous injection of propofol (100 mg kg^−1^). After disinfection with 2% iodine, a 5 mm diameter hole was drilled at the center of the skull, and the substrates were implanted into each hole with the NSCs seeded surface facing the brain tissue. Absorbable gelatin sponges were placed in the defect area to prevent substrate movement. Finally, the scalps were closed with sutures, and the animals were returned to their home cages. After 1 day, the mice were exposed to a rotating magnetic field (300 rpm) at a distance of 20 mm from the NdFeB magnet. The daily stimulation duration was 20 min for 7 days. Time‐matched control NSCs were kept without rotating magnetic field excitation during the same period. All mice were sacrificed 1 week after implantation and the brain tissues that contained the substrates were fixed in 10% formalin. Histological analyses were performed by an independent investigator using H&E staining, Nissl staining, and immunofluorescence staining. The quantitative mean immunofluorescence intensity of Tuj1 or MAP2 was analyzed in each group using Image J software. All animal experiments were approved by the Animal Care Committee of Qilu Hospital of Shandong University. All procedures were conducted in accordance with the guidelines of the Ethics Committee of Qilu Hospital of Shandong University (DWLL‐2019‐24).

### Statistical Analysis

Data are presented as the mean ± standard deviation. GraphPad Instant software (GraphPad, USA) was used for statistical analysis. One‐way analysis of variance was applied to determine the statistical significance of the differences. Differences were considered statistically significant at *p* < 0.05 (*p < 0.05, **p < 0.01, ***p < 0.001).

## Conflict of Interest

The authors declare no conflict of interest.

## Supporting information

Supporting Information

Supplemental Movie 1

Supplemental Movie 2

Supplemental Movie 3

Supplemental Movie 4

Supplemental Movie 5

Supplemental Movie 6

Supplemental Movie 7

## Data Availability

Research data are not shared.
