# Supplementary material for: Gold Nanostrip Array‐Mediated Wireless Electrical Stimulation for Accelerating Functional Neuronal Differentiation
Source: Adv Sci (Weinh). 2022 May 26;9(22):2202376. doi: 10.1002/advs.202202376 (PMC9353484; doi:10.1002/advs.202202376)
Supplement: Supplementary file 1 — Supporting Information [file ADVS-9-2202376-s004.pdf]

ADVANCED SCIENCE
Open Access

Supporting Information

for *Adv. Sci.*, DOI 10.1002/advs.202202376

Gold Nanostrip Array-Mediated Wireless Electrical Stimulation for Accelerating Functional Neuronal Differentiation

Hongru Yang, Yue Su, Zhaoyang Sun, Baojin Ma, Feng Liu, Ying Kong, Chunhui Sun, Boyan Li, Yuanhua Sang, Shuhua Wang, Gang Li, Jichuan Qiu, Chao Liu*, Zhaoxin Geng* and Hong Liu**

Supporting Information

Gold nanostrip array-mediated wireless electrical stimulation for accelerating functional neuronal differentiation

Hongru Yang, Yue Su, Zhaoyang Sun, Baojin Ma, Feng Liu, Ying Kong, Chunhui Sun, Boyan Li, Yuanhua Sang, Shuhua Wang, Gang Li, Jichuan Qiu, Chao Liu*, Zhaoxin Geng*, and Hong Liu**

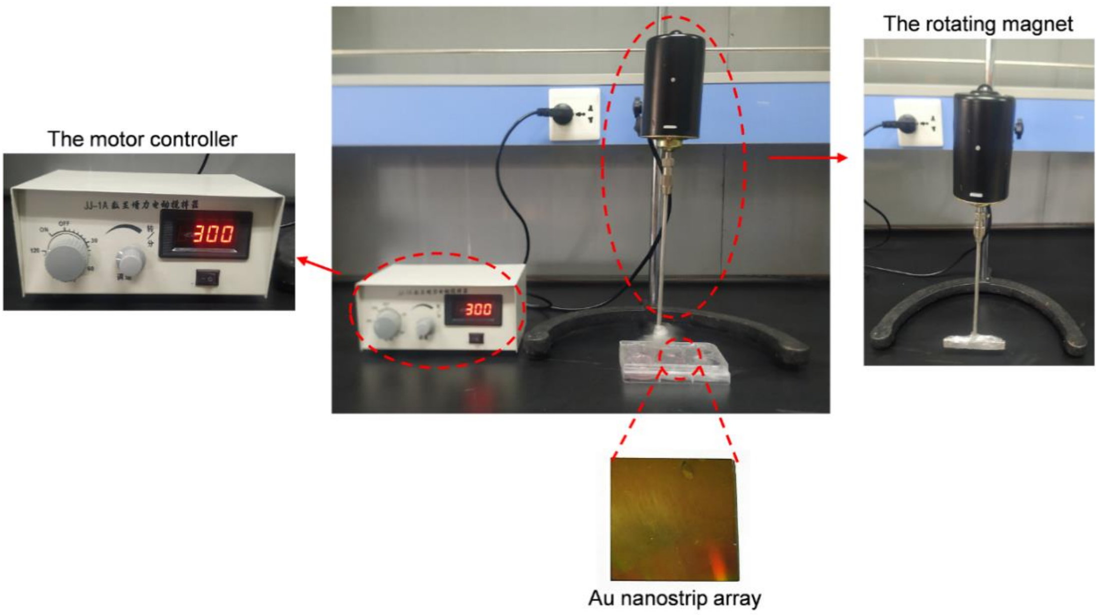

Figure S1. Experimental setup for generation of wireless electrical stimulation to NSCs.

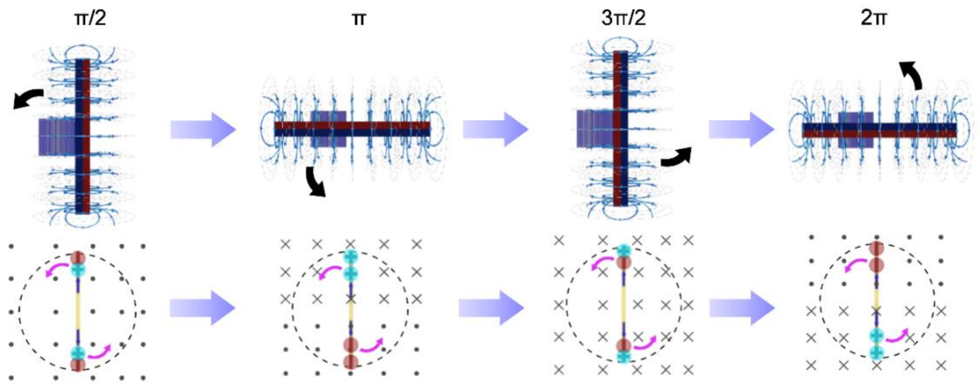

Figure S2. The working principle based on the law of electromagnetic induction. As the magnetic field rotated, the magnetic induction lines were constantly cut by Au nanostrips, creating positive and negative charges at each end of the Au nanostrip.

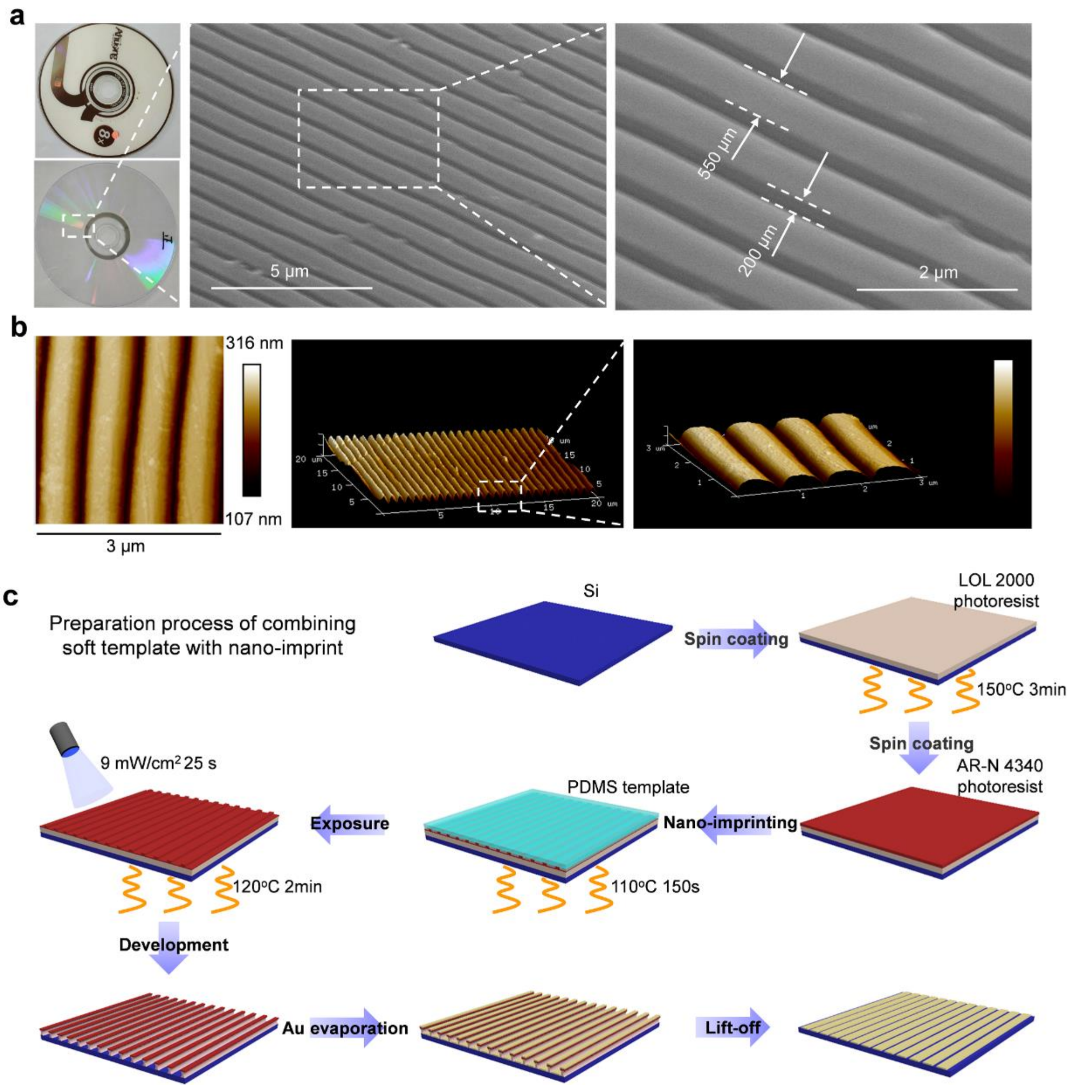

Figure S3. (a) Optical images and SEM images of the PC layer in cheap DVD. (b) AFM images of the PC layer in cheap DVD. (c) Fabrication process of the Au nanostrips array by NIL using cheap DVD as the initial template.

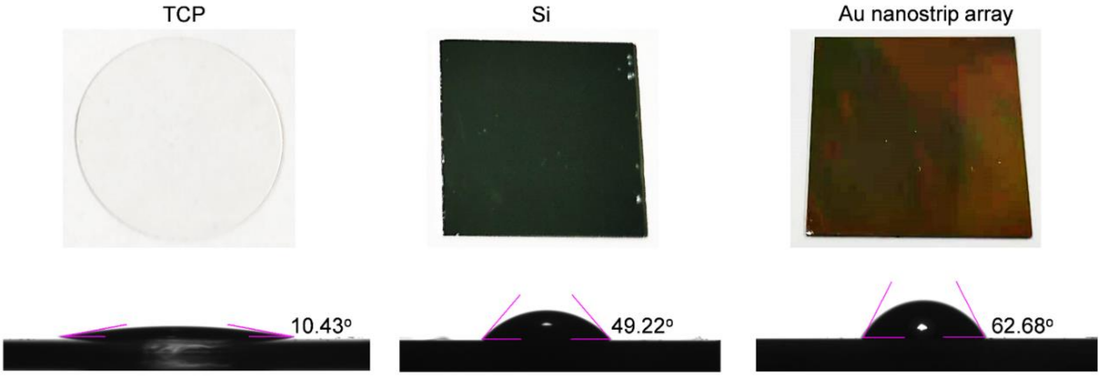

Figure S4. Optical images and water contact angle measurements of different substrates (TCP, Si, or Au nanostrips array).

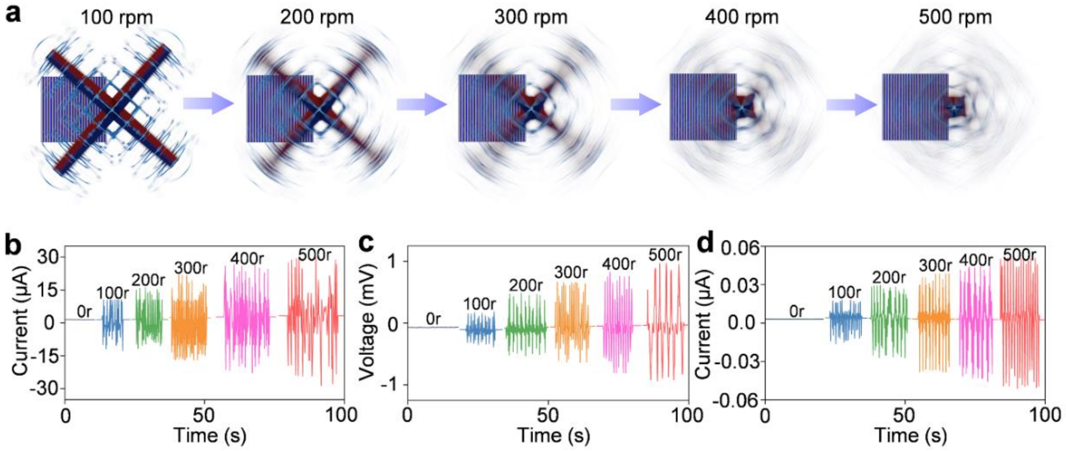

Figure S5. (a) Schematic diagram of rotating magnetic field at different speeds (100, 200, 300, 400, or 500 rpm). (b) The generated induced currents on the Au nanostrip array when the magnet was rotated at 0, 100, 200, 300, 400, or 500 rpm. And the generated induced (c) voltages and (d) currents on the Si when the magnet was rotated at 0, 100, 200, 300, 400, or 500 rpm.

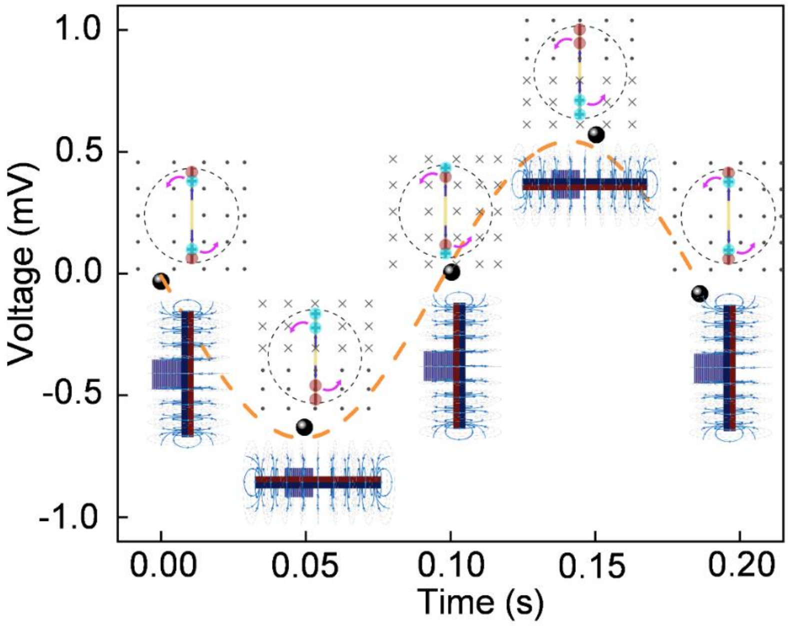

Figure S6. Fitting analysis of induction output electrical signal measured experimentally. The results show the induction output electrical signal conformed to the law of sine function, and the sinusoidal pulse signal period was 0.02s, which is consistent with the rotation frequency of the rotating magnet (300 rpm).

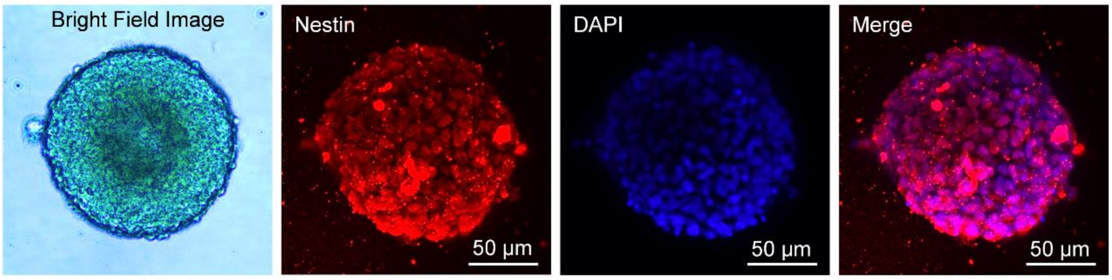

Figure S7. Bright field image and immunofluorescence staining of the third-passage neurosphere. Nestin were stained into red. Cell nucleus were stained into blue with DAPI.

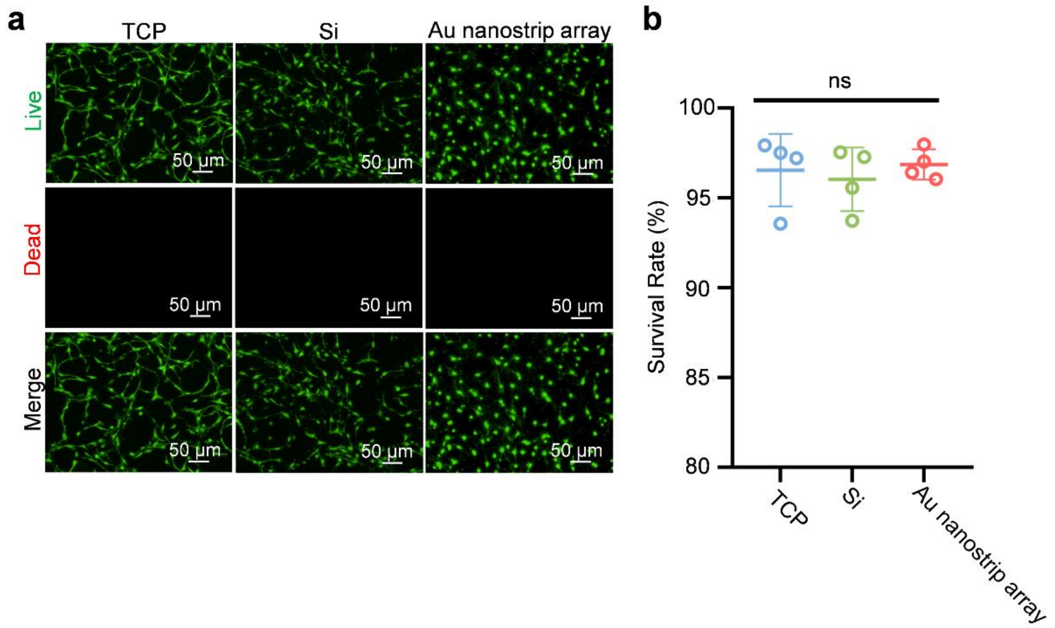

Figure S8. (a) Live/dead staining (Green (calcein-AM) for live cells and red (propidium iodide) for dead cells) and (b) the quantified cell survival rate assay of NSCs cultured on different substrates (TCP, Si, or Au nanostrip array) for 3 days. Data are presented as the mean ± standard deviation. $^{ns}p > 0.05$.

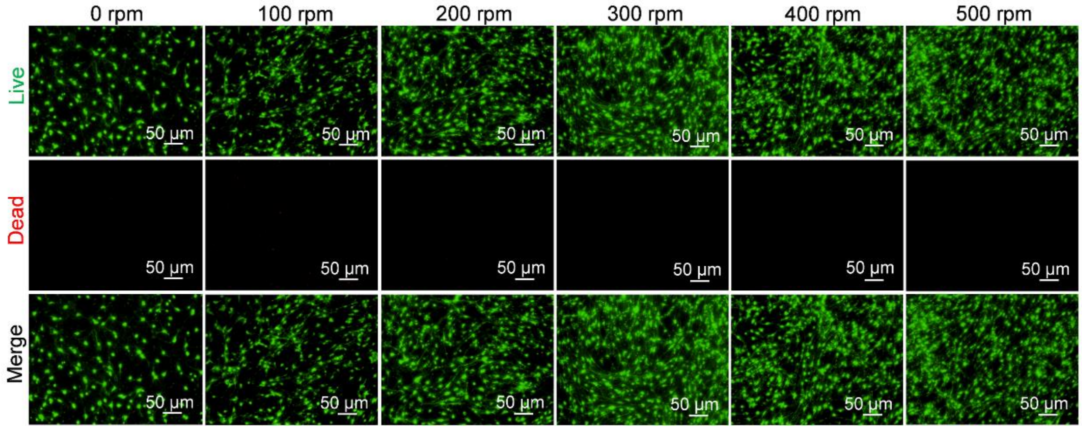

Figure S9. Live/dead staining of NSCs after culture on Au nanostrip array for 3 days with the magnet rotating at 0, 100, 200, 300, 400, or 500 rpm. Green (calcein-AM) for live cells and red (propidium iodide) for dead cells.

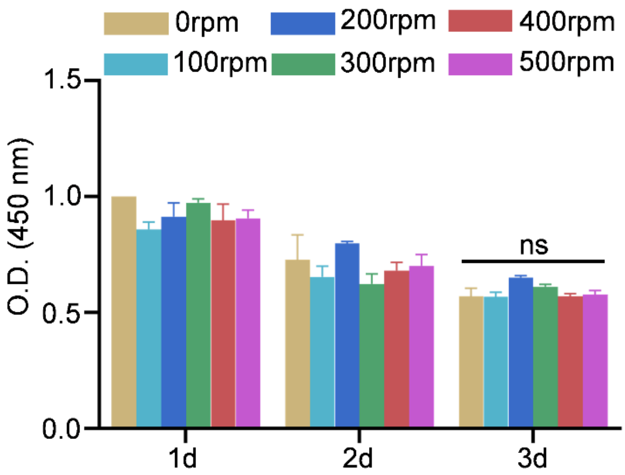

Figure S10. Viability of NSCs after culture on Au nanostrip array with the magnet rotating at 0, 100, 200, 300, 400, or 500 rpm for 1, 2, or 3 days. Data are presented as the mean ± standard deviation (n = 4). $^{ns}p > 0.05$.

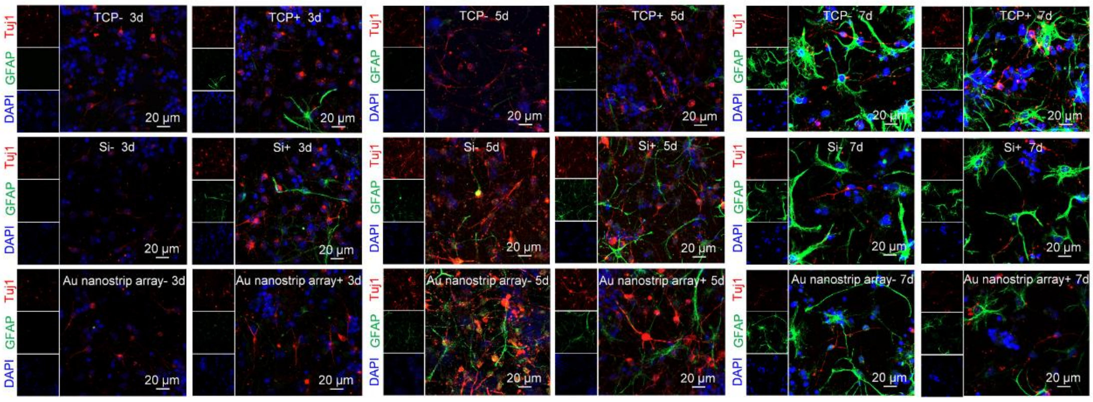

Figure S11. Confocal microscope images of NSCs seeded on different substrates (TCP, Si, or Au nanostrip array) and cultured without or with rotating magnetic field (300 rpm) for 3, 5, or 7 days. Tuj1 and GFAP were stained into red and green, respectively. Cell nucleus were stained into blue with DAPI.

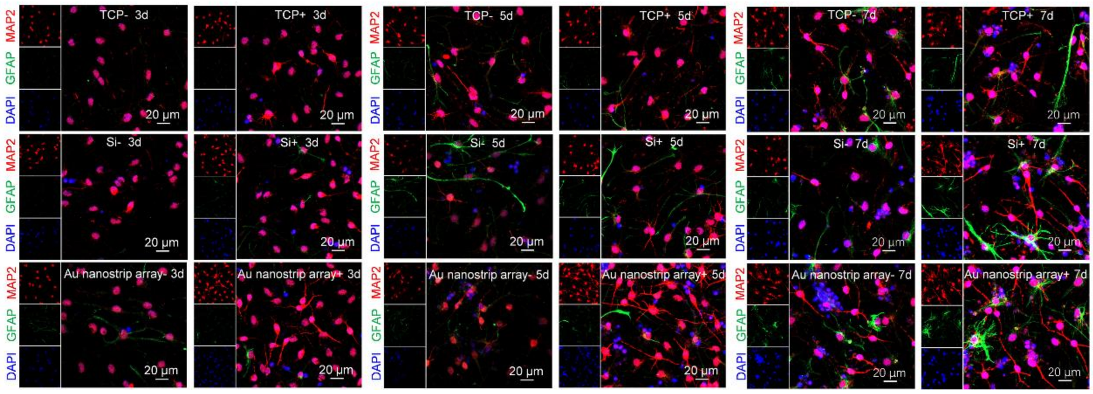

Figure S12. Confocal microscope images of NSCs seeded on different substrates (TCP, Si, or Au nanostrip array) and cultured without or with rotating magnetic field (300 rpm) for 3, 5, or 7 days. MAP2 and GFAP were stained into red and green, respectively. Cell nucleus were stained into blue with DAPI.

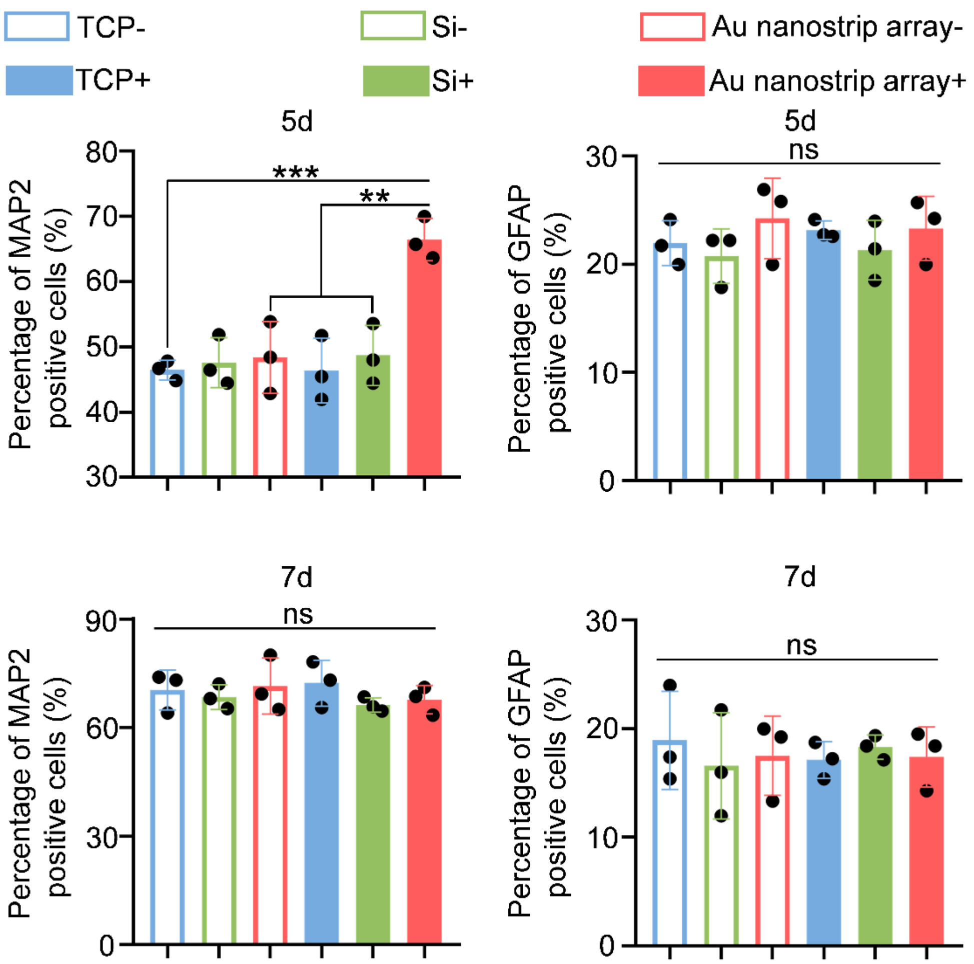

Figure S13. The percentage of MAP2 positive cells or GFAP positive cells was calculated and normalized to nuclei in the NSC populations seeded on different substrates (TCP, Si, or Au nanostrip array) and cultured without or with rotating magnetic field (300 rpm) for 5 or 7 days. Quantitative analysis data are presented as the mean ± standard deviation (n = 3); $^{ns}p > 0.05$, $^{**}p < 0.01$.

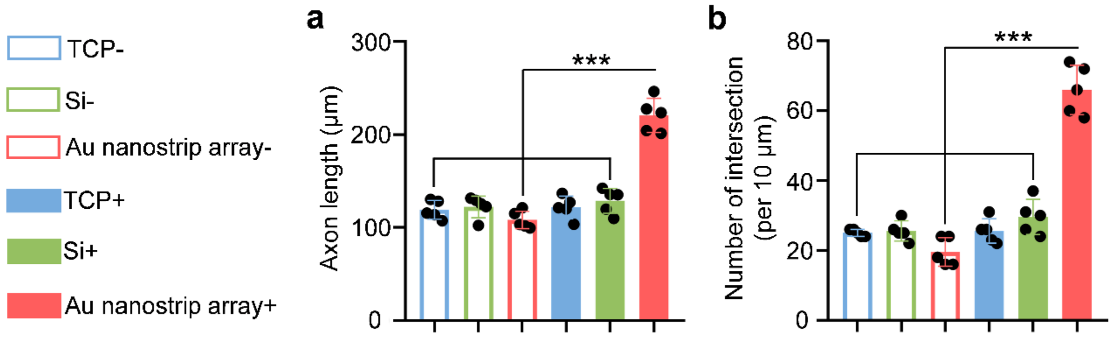

Figure S14. Statistical analyses of the axon length (a) and total neurite branching (b) of the NSCs seeded on different substrates (TCP, Si, or Au nanostrip array) and cultured without or with rotating magnetic field (300 rpm) for 5 days according to the sholl analysis that presented in Figure 3f. Quantitative analysis data are presented as the mean ± standard deviation (n = 5). ***$p < 0.001$.

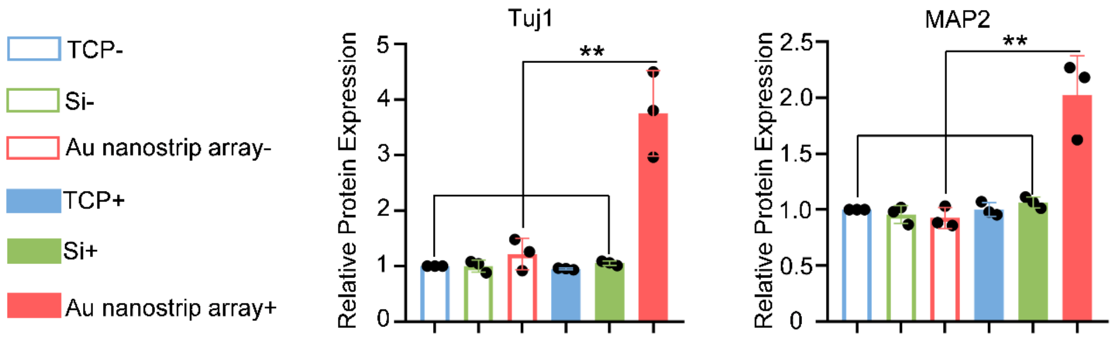

Figure S15. The semi-quantitative analysis of the Tuj1 and MAP2 protein expression of NSCs seeded on TCP, Si, or Au nanostrip array and cultured without or with rotating magnetic field (300 rpm) on day 5. Glyceraldehyde 3-phosphate dehydrogenase (GAPDH) was used as the housekeeping gene. Quantitative analysis data obtained using Image J software are presented as mean ± standard deviation ($n = 3$); $^{**}p < 0.01$.

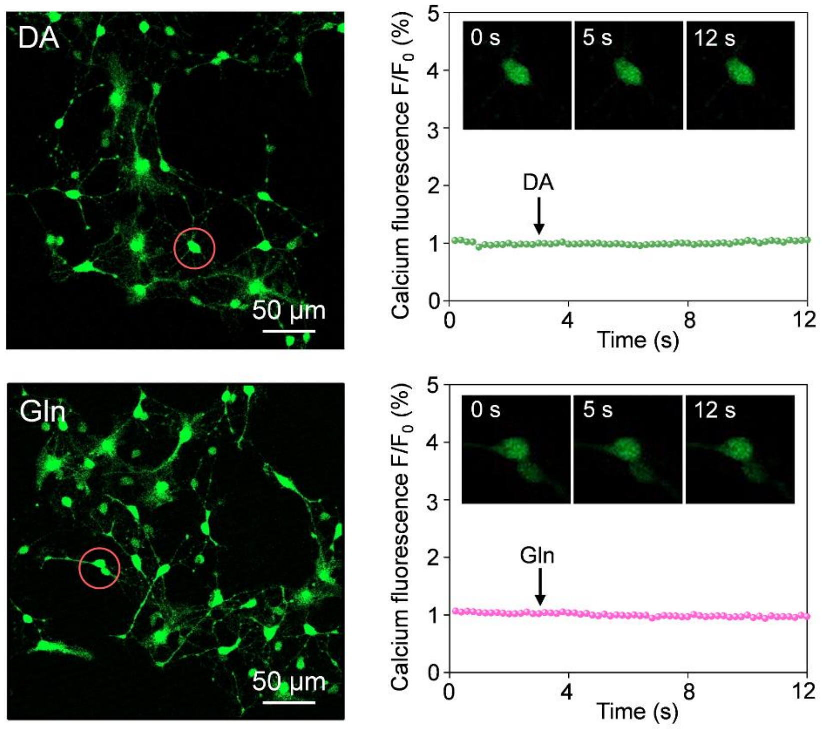

Figure S16. Calcium dynamics within the differentiated neurons seeded on Au nanostrip array and cultured with rotating magnetic field (300 rpm) for 5 days (calcium, green). Left, images of neurons after calcium dye (Fluo-4 AM) loading. Right, the time-series imaging of relative fluorescence intensity change %ΔF/F for individual neurons after stimulation by the neurotransmitters (dopamine (DA) and l-glutamate (Gln)). The inset shows the fluorescence intensity expression of the cell body in the selected region at different time points.

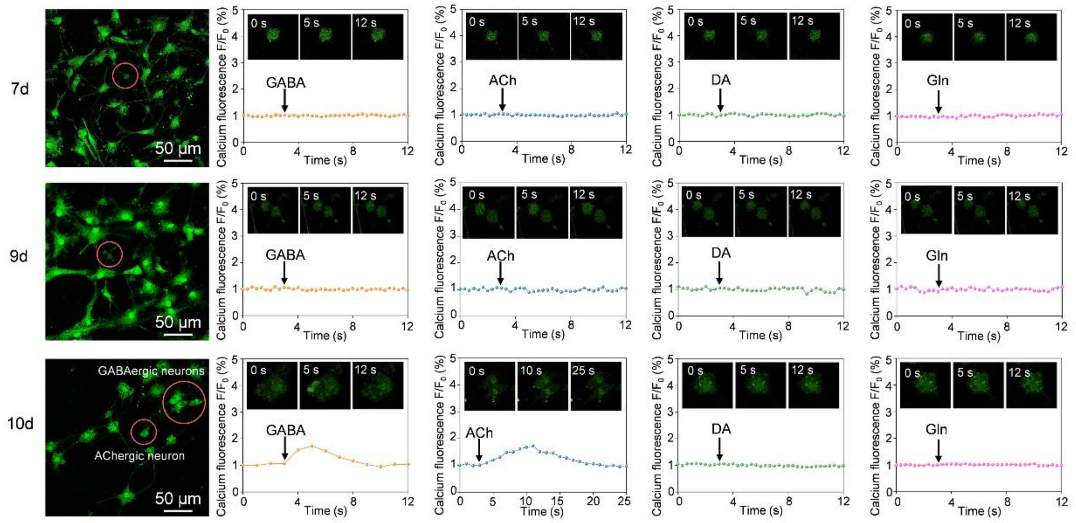

Figure S17. Calcium dynamics within the differentiated neurons seeded on TCP for 7, 9, or 10 days (calcium, green). Left, images of neurons after calcium dye (Fluo-4 AM) loading. Right, the time-series imaging of relative fluorescence intensity change %ΔF/F for individual neurons after stimulation by the neurotransmitters (g-aminobutyric acid (GABA), acetylcholine (ACh), dopamine (DA), or l-glutamate (Gln)). The inset shows the fluorescence intensity expression of the cell body in the selected region at different time points.

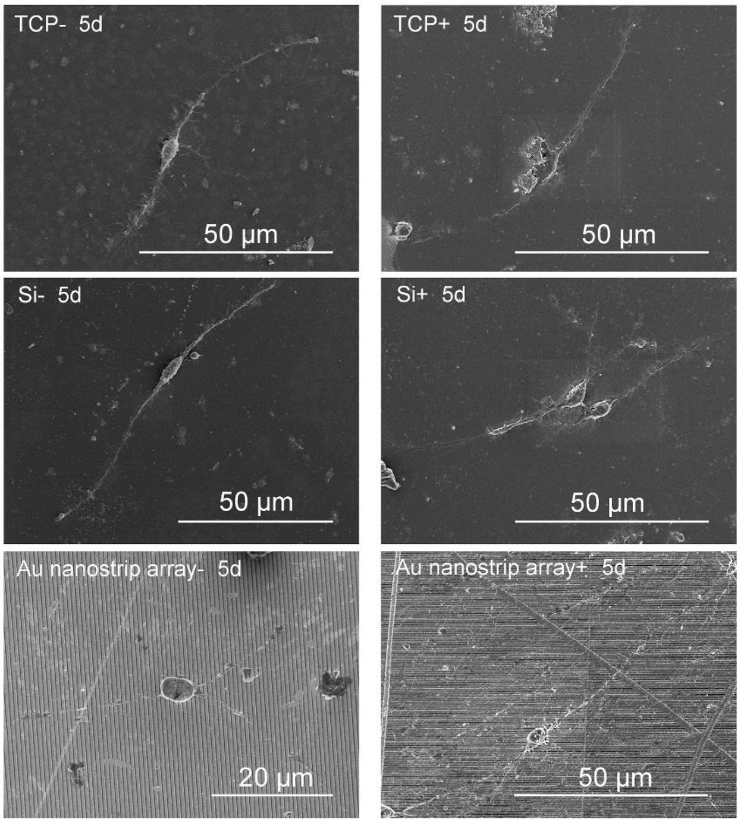

Figure S18. SEM images of the differentiated neurons on different substrates (TCP, Si, or Au nanostrip array) and cultured without or with rotating magnetic field (300 rpm) for 5 days.

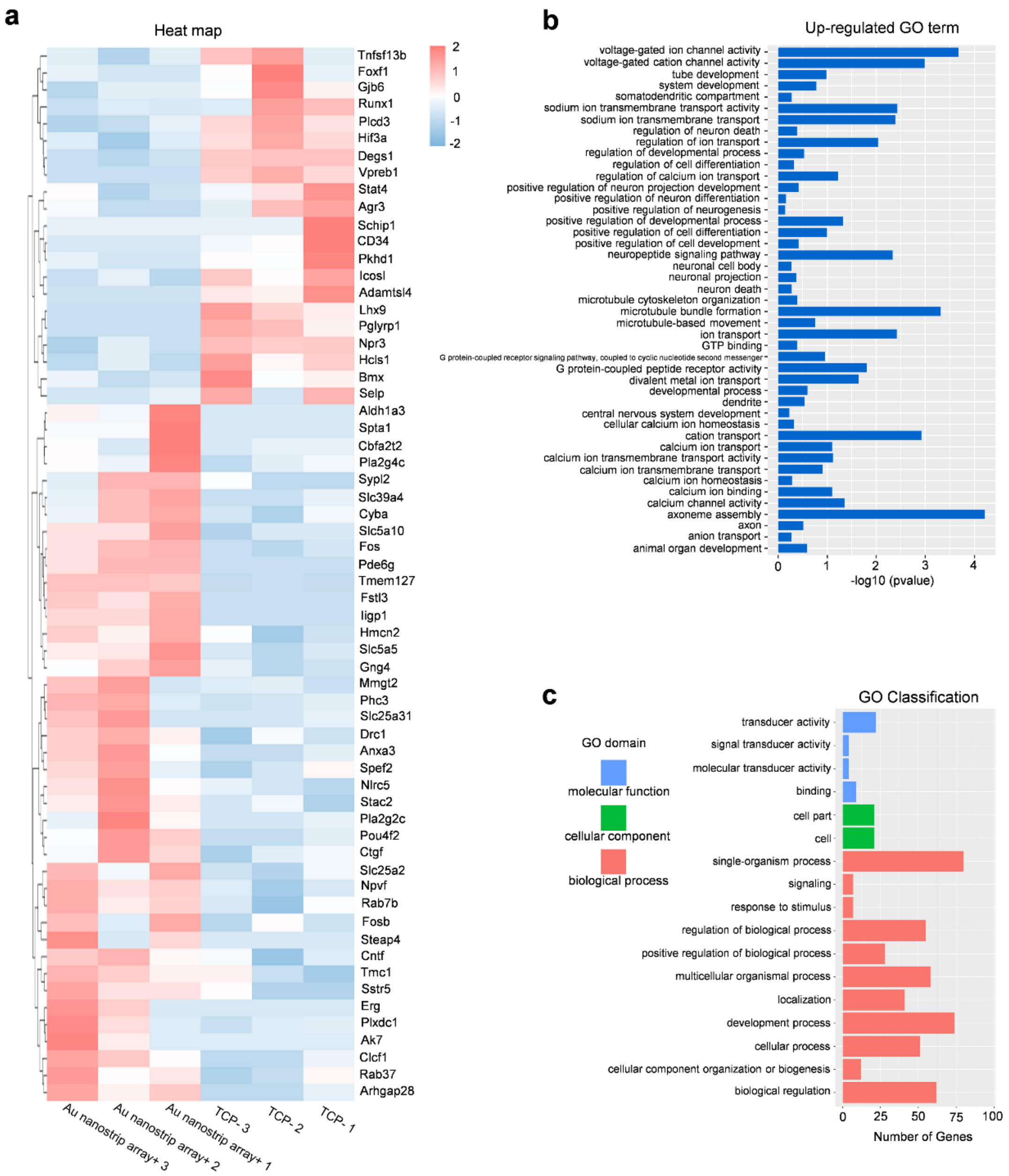

Figure S19. (a) Heat map illustrating the differentially expressed genes between the Au nanostrip array+ and TCP- groups. Each lane corresponds to an independent biological sample. (b) GO analysis of the upregulated expressed genes in Au nanostrip array+ group compared with TCP- group. (c) GO enrichment classification of differentially expressed genes between the Au nanostrip array+ and TCP- groups.

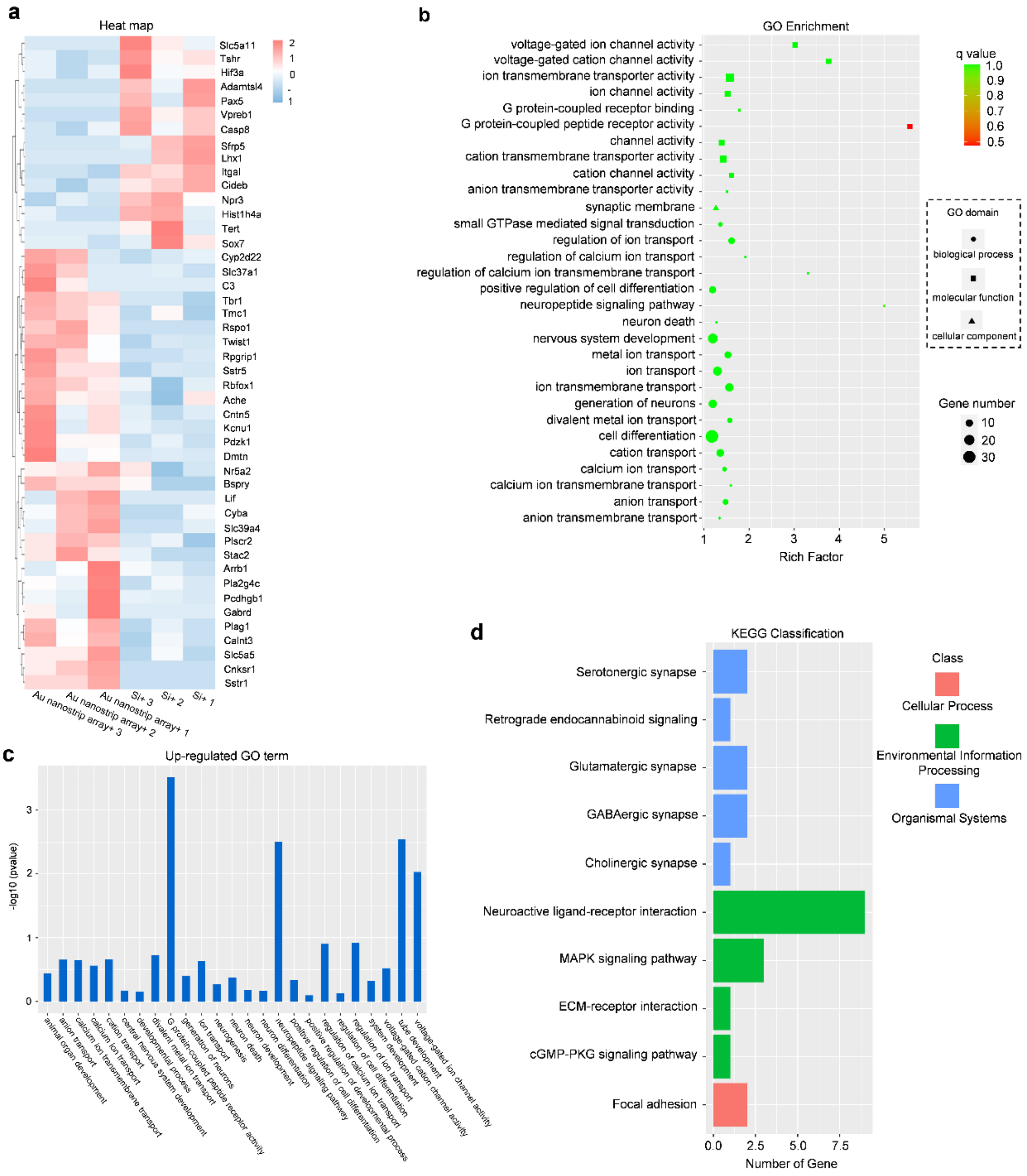

Figure S20. (a) Heat map illustrating the differentially expressed genes between the Au nanostrip array+ and Si+ groups. Each lane corresponds to an independent biological sample. (b) GO functional enrichment analysis of differentially expressed genes between the Au nanostrip array+ and Si+ groups. (c) GO analysis of the upregulated expressed genes in Au nanostrip array+ group compared with Si+ group. (d) KEGG pathways classification of differentially expressed genes between the Au nanostrip array+ and Si+ groups.

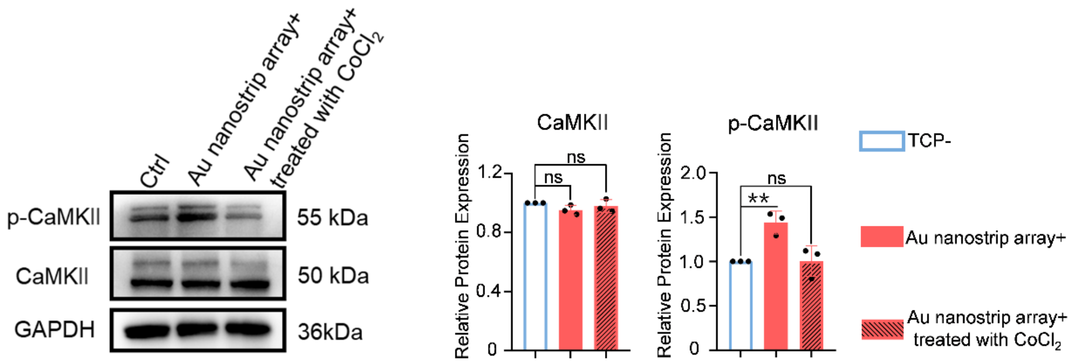

Figure S21. Western blot analysis of the CaMKII and p-CaMKII in NSCs that cultured on Au nanostrip array and treated without or with treated with 3 mM $CoCl_2$ before rotating magnetic field was applied on day 5. GAPDH was used as the housekeeping gene. Quantitative analysis data are presented as the mean ± standard deviation (n = 3). $^{ns}p > 0.05$, $^{**}p < 0.01$.

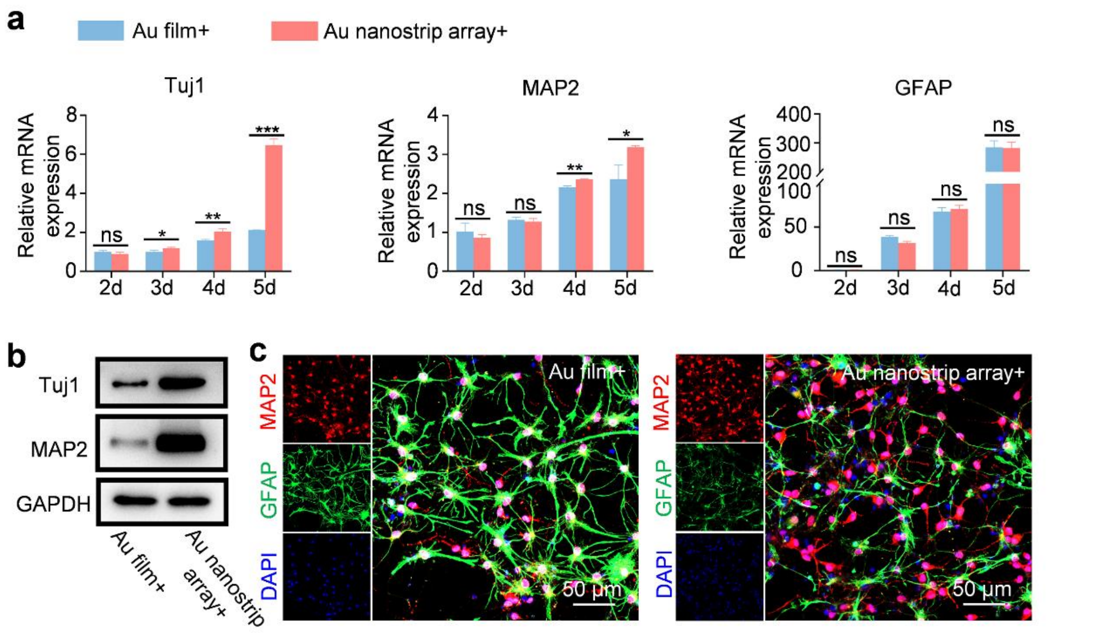

Figure S22. (a) RT-qPCR analysis of the expression of neural related genes (Tuj1, MAP2, GFAP) for NSCs seeded on different substrates (Au film, or Au nanostrip array) and cultured without or with rotating magnetic field (300 rpm) for 2, 3, 4, or 5 days. Data are presented as the mean ± standard deviation (n = 3). $^{ns}p > 0.05$, $*p < 0.05$, $**p < 0.01$, $***p < 0.001$. (b) Western blot analysis of the Tuj1 and MAP2 expression for NSCs seeded on different substrates (Au film, or Au nanostrip array) and cultured without or with rotating magnetic field (300 rpm) at day 5. GAPDH serves as a housekeeping gene. (c) Confocal microscope images of NSCs seeded on different substrates (Au film, or Au nanostrip array) and cultured without or with rotating magnetic field (300 rpm) at day 5. MAP2 and GFAP were stained into red and green, respectively. Cell nucleus were stained into blue with DAPI.

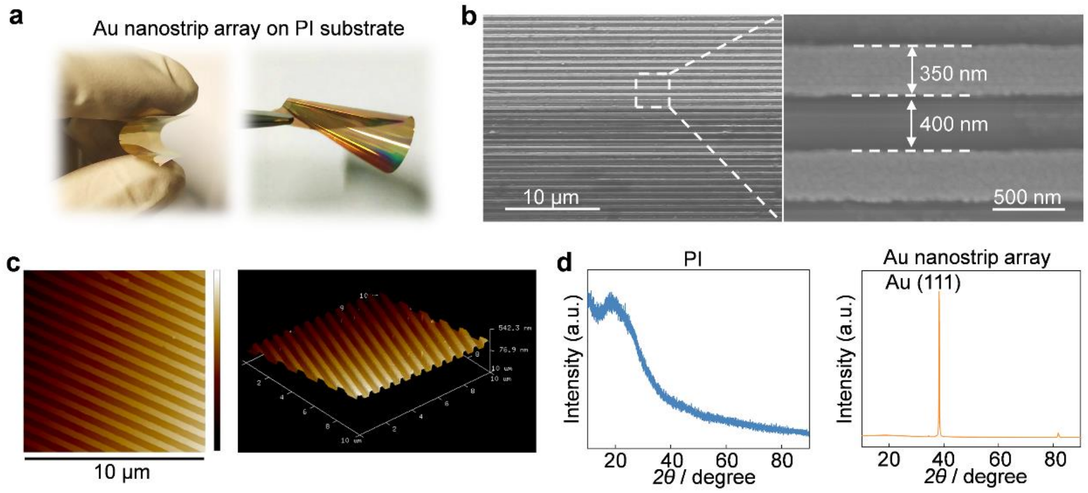

Figure S23. (a) Optical images of Au nanostrip array that prepared on PI. (b) SEM images of Au nanostrip array that prepared on PI. (c) AFM images Au nanostrip array that prepared on PI. (d) X-ray diffraction pattern of PI (left) and Au nanostrip array that prepared on PI (right).

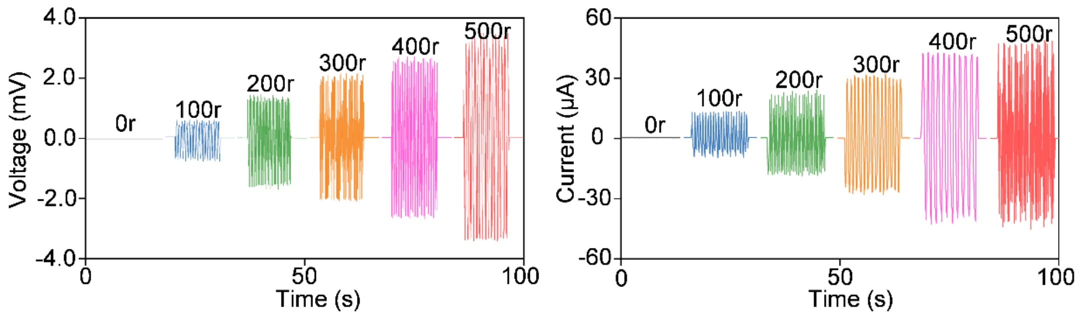

Figure S24. The generated induced voltages and currents on the Au nanostrip array that prepared on PI when the magnet was rotated at 0, 100, 200, 300, 400, or 500 rpm.

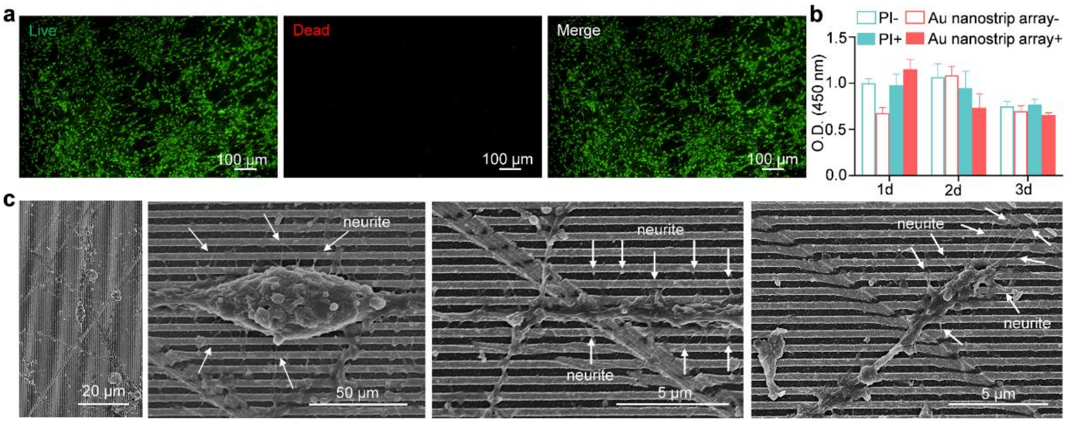

Figure S25. (a) Live/dead staining of NSCs after culture on Au nanostrip array that prepared on PI for 3 days with the rotating magnet field (300 rpm). Green (calcein-AM) for live cells and red (propidium iodide) for dead cells. (b) Viability of NSCs seeded on different substrates (Au film, or Au nanostrip array) and cultured without or with rotating magnetic field (300 rpm) for 1, 2, or 3 days. Data are presented as the mean ± standard deviation (n = 3). (c) SEM images of differentiated NSCs on Au nanostrip array with rotating magnetic fields (300 rpm) for 3 days. Arrows point to neurites.

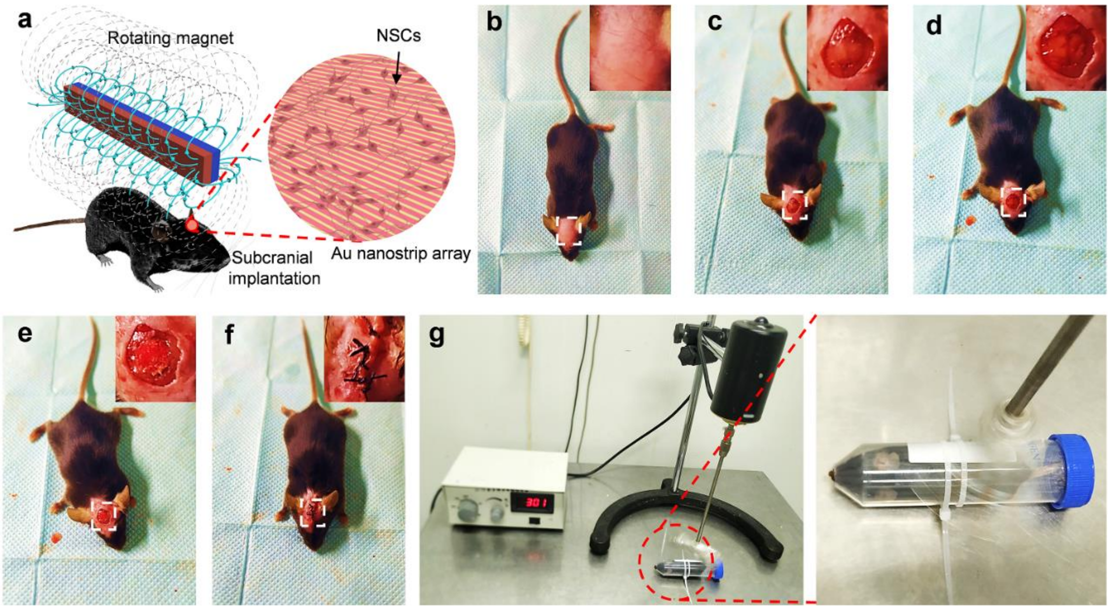

Figure S26. Process of Au nanostrip array accelerating neuronal differentiation of NSCs *in vivo*. (a) Schematic diagram of Au nanostrip array accelerating neuronal differentiation of NSCs *in vivo*. (b) The 6-week-old C57 BL/6 mouse was anesthetized and the hair in the surgical area of the head was shaved clean. (c) Cut open the scalp to expose the skull. (d) A 5 mm diameter hole was drilled on the center of the skull and the dura mater was further destroyed. (e) The Au nanostrip array seed with NSCs was implanted in 5 mm skull hole and attached the cerebral cortex in C57 mouse. (f) Absorbable gelatin sponge was placed in the defect area for compression to stop the bleeding and then sutured. (g) After one day, mouse was exposed to rotating magnetic field (300 rpm) at distance of 20 mm from the NdFeB magnet. The daily stimulation duration was 20 min for 7 days. The 50 mL tube with the bottom opening serves as a simple temporary fixing device.

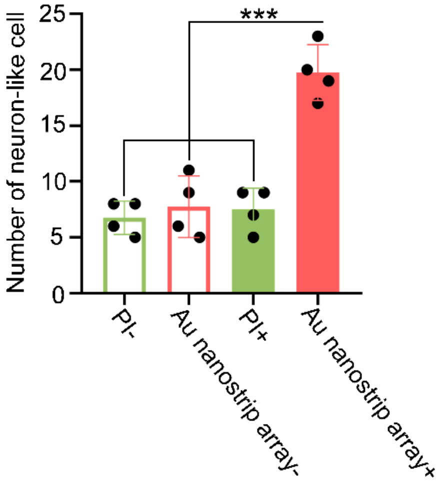

Figure S27. Statistical analyses of the number of neuron-like cells in each group by combining the results of Hematoxylin and eosin (H&E) staining and Nissl staining on day 7. The number of neuron-like cells in each group are presented as the mean ± standard deviation (n = 4). ***$p < 0.001$.

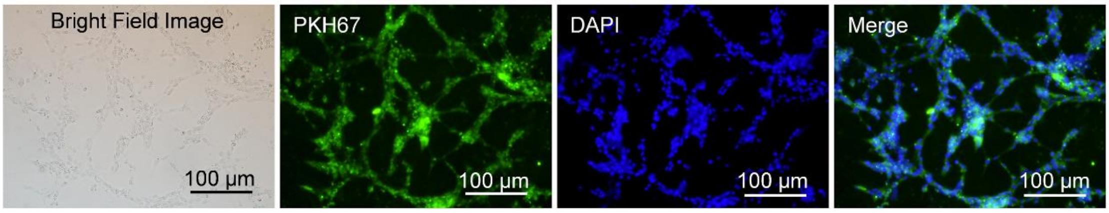

Figure S28. Bright field image and fluorescence images of NSCs that pre-labeled with PKH67 seed on TCP for 24 h. Cell membranes were stained with PKH67 (green), and cell nucleus were stained with DAPI (blue).

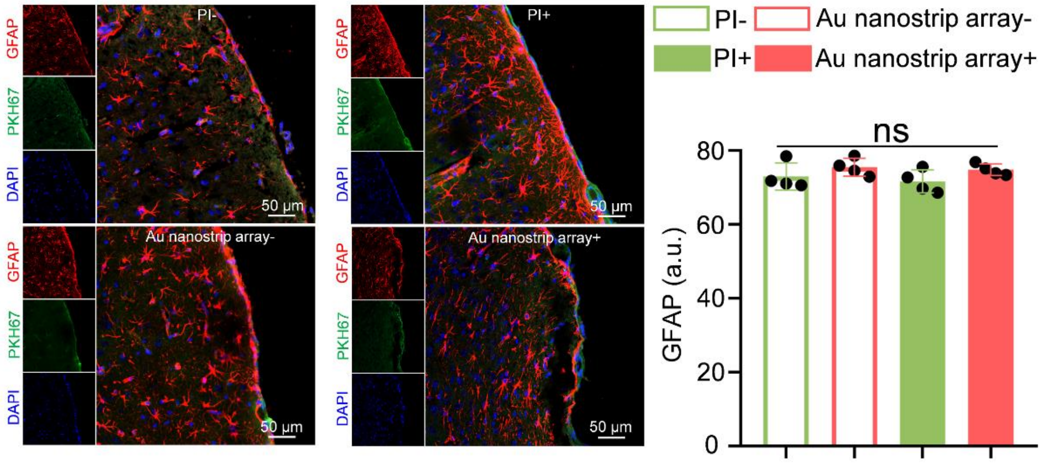

Figure S29. Immunofluorescent images of the cerebral cortex section obtained at the interface where substrates (PI, or Au nanostrip array) inoculated with NSCs were applied without or with the rotating magnetic field (300 rpm) at day 7. GFAP and cell membrane were stained into red and green, respectively. Cell nucleus were stained into blue with DAPI. The quantitative mean immunofluorescence intensity of GFAP in each group are presented as the mean ± standard deviation (n = 4). $^{ns}p > 0.05$.

Table S1. Sequences of RT-qPCR primers. All primers purchased from BioSune Biotechnology (ShangHai) Co., Ltd. and the primer sequence of the target genes are displayed below.

Gene	Forward primers (5'-3')	Reverse primers (5'-3')
β-actin	GGCTGTATTCCCCTCCATCG	CCAGTTGGTAACAATGCCATGT
Tuj1	TATGAAGATGATGACGAGGAATCG	TACAGAGGTGGCTAAAATGGGG
Map2	TGGAGGAAGCAGCAAGTG	AGGGAGGATGGAGGAAGG
GFAP	CCAAGCCAAACACGAAGCTAA	CATTTGCCGCTCTAGGGACTC
